# The role of glutamate in neuronal ion homeostasis: A case study of spreading depolarization

**DOI:** 10.1371/journal.pcbi.1005804

**Published:** 2017-10-12

**Authors:** Niklas Hübel, Mahshid S. Hosseini-Zare, Jokūbas Žiburkus, Ghanim Ullah

**Affiliations:** 1 Department of Physics, University of South Florida, Tampa, Florida, United States of America; 2 Department of Biology and Biochemistry, University of Houston, Houston, Texas, United States of America; École Normale Supérieure, College de France, CNRS, FRANCE

## Abstract

Simultaneous changes in ion concentrations, glutamate, and cell volume together with exchange of matter between cell network and vasculature are ubiquitous in numerous brain pathologies. A complete understanding of pathological conditions as well as normal brain function, therefore, hinges on elucidating the molecular and cellular pathways involved in these mostly interdependent variations. In this paper, we develop the first computational framework that combines the Hodgkin–Huxley type spiking dynamics, dynamic ion concentrations and glutamate homeostasis, neuronal and astroglial volume changes, and ion exchange with vasculature into a comprehensive model to elucidate the role of glutamate uptake in the dynamics of spreading depolarization (SD)—the electrophysiological event underlying numerous pathologies including migraine, ischemic stroke, aneurysmal subarachnoid hemorrhage, intracerebral hematoma, and trauma. We are particularly interested in investigating the role of glutamate in the duration and termination of SD caused by K^+^ perfusion and oxygen-glucose deprivation. Our results demonstrate that glutamate signaling plays a key role in the dynamics of SD, and that impaired glutamate uptake leads to recovery failure of neurons from SD. We confirm predictions from our model experimentally by showing that inhibiting astrocytic glutamate uptake using TFB-TBOA nearly quadruples the duration of SD in layers 2-3 of visual cortical slices from juvenile rats. The model equations are either derived purely from first physical principles of electroneutrality, osmosis, and conservation of particles or a combination of these principles and known physiological facts. Accordingly, we claim that our approach can be used as a future guide to investigate the role of glutamate, ion concentrations, and dynamics cell volume in other brain pathologies and normal brain function.

## Author summary

Pathological conditions such as seizure, migraine, traumatic brain injury, and stroke are associated with extreme changes in ion concentrations and glutamate, cell swelling, and heavy exchange of matter between neurons, glia, and vasculature. However, current experimental tools are capable of measuring only a few of these variables, which necessitates the development of biophysically relevant models. This study provides a comprehensive computational framework derived from first physical principles and physiological facts that enables us to investigate a wide range of key variables in SD caused by K^+^ perfusion and oxygen-glucose deprivation. While we use SD as an example, our approach can be extended to other pathological conditions and normal brain function.

## Introduction

Spreading depolarization (SD) is a self-propagating wave characterized by a near-complete breakdown of transmembrane ion gradients in cells, sustained depolarization in individual neurons, and swelling of neuronal and glia cells [1, 2, 3, 4, 5]. It is now well accepted that SD is relevant to many neurological disorders. Several studies have shown that SD is the pathophysiological correlate of the symptoms of migraine aura [6, 7, 8, 9, 10, 11], and occurs frequently in acutely injured brain caused, for example, by ischemic stroke, aneurysmal subarachnoid hemorrhage, and trauma [12, 3, 13, 14, 4, 5]. Several clinical studies by COSBID group [15] and others suggest that SD mediates cortical lesion development and secondary brain damage in patients with acute brain injury, impairs clinical recovery, and triggers new deficits [12, 13, 3]. Furthermore, significant evidence indicates that SD and epileptic seizures might have some shared mechanisms [16, 17, 18, 19].

The local processes during SD are understood as the interplay of neurons, astrocytes, and the vascular system. The neuron releases large amounts of K^+^ and glutamate into the extracellular space (ECS) together with significant drop in extracellular Ca^2+^, Na^+^, Cl^−^, and pH when it depolarizes. Consequently, SD is accompanied by significant extracellular K^+^ and glutamate accumulation, activation of N-methyl-D-aspartate (NMDA) receptors, a general loss of ion homeostasis, and cytotoxic edema [20, 21, 22, 23, 4, 5, 24, 25, 26, 27].

Excitotoxicity and SD are largely overlapping phenomena. Glutamate is of particular interest because of its role in excitotoxicity and its synchronous extracellular rise with the onset of SD [13, 28, 27]. Activation of NMDA receptors by glutamate triggers the release of further glutamate and K^+^ that will diffuse to neighboring cells thus causing the propagation and sustainment of SD. This hypothesis is backed by significant evidence of glutamate receptors antagonists inhibiting SD. Slices experiments showed that ischemic cells with NMDA and non-NMDA receptors blocked, did not exhibit the fatal form of SD [26, 29]. An NMDA receptor antagonist, Ketamine was shown to inhibit SD in swine cortex [30] and reduced the number of SD incidences in patients [29, 31].

Astrocytes and vasculature are other key players regulating many aspects of SD [4, 14]. In addition to coordinating matter transport between vasculature and neurons and playing a major role in the observed metabolic and hemodynamics effects that are key to our understanding of numerous neurovascular diseases, astrocytes protect against SD initiation due to their high capacity for K^+^ and glutamate uptake [14]. Increasing the expression of astrocytic glutamate transporters reduces the infarct volumes following ischemia [32] and protects against the onset of ischemia-induced SD [33]. Astrocytic swelling together with changes in neuronal volume can exacerbate SD and may lead to severe brain damage [34, 35]. In astrocytes, volume–activated anion channels may release large amounts of glutamate leading to excitotoxic damage [36]. The knockouts of aquaporin 4 channels that mediate astrocytic swelling [37], protect against ischemia [38].

To summarize, SD is accompanied by an array of immense changes from molecular to network level. A better understanding of SD and a spectrum of related pathologies, therefore, hinges on elucidating the pathways involved in these changes. However, existing techniques are too limited to investigate all these pathways. To overcome this void, we develop a comprehensive model that takes into account these key variables to quantify the role of glutamate dynamics in SD. We are particularly interested in SD caused by K^+^ perfusion and oxygen glucose–deprivation (OGD). The model equations are either derived purely from first physical principles of electroneutrality, osmosis, and conservation of particles, or by a phenomenological combination of these principles and known physiological facts. Our model is successful in explaining experimental results about the role of glutamate in SD. We confirm the predictions of our model by showing that astrocytic glutamate transporters blocker (2S, 3S)-3-[3-[4-(trifluoromethyl) benzoylamino]benzyloxy]aspartate (TFB-TBOA) significantly elongates the duration of SD in cortical slices from 15-24 days old rats. While our discussion is focussed on glutamate, the model can be used to explore the role of other key pathways and swelling in the dynamics of SD. Furthermore, the framework can be applied to investigate the role of ion concentrations, glutamate, and cellular volume dynamics in other pathological conditions and normal brain function.

Numerous single neuron models for investigating SD have been developed. The phenomenon is rather generic and is found in models with great physiological details [39, 40, 41, 42] as well as in simplified HH based descriptions of the neuron [43, 44, 45, 46, 47, 39, 48, 49]. With the help of these models, thresholds for SD ignition and recovery can be assessed. In particular, it can be analyzed how energy and oxygen supply, morphological parameters, and blood pressure affect the course of SD, how SD can be prevented, and when it is non–recoverable [50, 44, 51, 52, 43, 53]. Only few of these models deal with swelling. Some incorporate neuronal swelling alone [54, 44, 43, 55], while one model [49] deals only with the astrocytic volume. Only two models Ref. [40, 56] include neuronal and astrocytic swelling simultaneously. The models in Refs. [57] and [58] for regular neuronal spiking and epileptic seizures respectively deal with astrocytic glutamate uptake with no ion concentration dynamics or swelling. The model in Ref. [58] does not include glutamate release from neurons during spiking. To our knowledge, no neuronal model (SD or otherwise) deals simultaneously with ion concentrations, neuronal and glial volume changes, and glutamate dynamics. As discussed above, the extreme changes and interdependence of these pathways during SD warrants a comprehensive computational framework encompassing all these key pathways—the subject of this paper.

## Methods

We use a single cell model that describes the electrical properties of the neuron and its ion dynamics. The biophysical mechanisms at work are gated channel dynamics, transmembrane ion fluxes and ion accumulation, ion regulation by K^+^/Na^+^–exchange pumps, glial K^+^ buffering, and ion exchange with an extracellular bath. These processes govern neural ion dynamics which in turn can induce osmotic cell swelling. For these parts of our model we employ a standard description based on earlier computational studies [45, 46, 53, 56, 59, 43, 60, 61, 44, 51, 62, 63, 64, 65].

To assess the interplay of ion dynamics and neurotransmitters, we add a range of glutamate–related processes to the model. This yields the first computational model that combines neural ion dynamics, neuronal and astrocytic swelling, and glutamate. There are enormous simplifications at work and we like to emphasize that the goal of this study is to unveil how glutamate affects ion dynamics and assess the relevance of the effects we find. Microscopic details regarding glutamate dynamics itself are beyond the scope of this work.

### Standard model for neural ion dynamics

Rate equations for the membrane potential of the neuron, gating variables for K^+^ and Na^+^ channels, ion concentrations inside the neuron, glia, and ECS, and volumes of the neuron, glia cell, and ECS are based on our previous work [45, 46, 53, 56, 59, 43, 60, 61, 44, 51, 63, 64]. These equations together with the modifications due to the inclusion of glutamate dynamics, and the morphology used in this model are described in S1 Text. Here we outline the details about modeling the glutamate homeostasis.

### Glutamate–related processes

Glutamate is a neurotransmitter that is released into the cleft of a synaptic connection when the presynaptic, i.e. signal–sending, neuron depolarizes. Glutamate binds to the NMDA and AMPA receptors of the postsynaptic neuron and can thereby initiate an action potential (AP). After binding to a receptor the transmitter is free again and can bind another time or diffuse into the ECS. Neurons and glia cells clear glutamate by taking it up from the cleft or from the ECS. For an overview of glutamate–related processes we refer the reader to reviews by Benarroch [66], and Kandel et al. [67] (see part III). Several components of the computational model presented in this section are adapted from various computational studies [68, 69, 70, 58, 71] and have been modified or extended for the application to SD.

#### Single cell model as local average

Spreading depolarization is an event of locally highly synchronous neural activity that involves nearly all synapses. Our single cell model shall provide a local average of this situation. Every synaptic connection belongs to two neurons and so does the amount of glutamate that is released into the cleft. So when glutamate is released into 10,000 synapses and then diffuses from the synaptic clefts into the ECS, we assume that only 50% of these glutamate molecules go into the ECS associated with our single neuron. Also only 50% of neural glutamate re–uptake belongs to our single neuron and similarly only 50% of glial glutamate uptake belongs to the glia compartment we model. We will also assume that the model neuron has 50% presynaptic and 50% postsynaptic connections.

We can now estimate the order of magnitude of glutamate release and the implied concentration in the ECS. During an action potential about 3,000 glutamate molecules are released into the synaptic cleft [72, 73, 74]. Since SD is such a highly synchronous event we assume this amount of glutamate release in all 10,000 synapses [75]. If all this glutamate would enter the ECS, there would be an increase in the extracellular glutamate concentration of about 0.0033 mM, assuming an ECS volume size of 7,500 *μ*m^3^. After a series of 20 action potentials we would have a concentration of about 0.066 mM. In spreading depression the ECS volume reduces by up to 75% leading to glutamate concentration of about 0.266 mM if no re–uptake is at work.

This shows that the ECS cannot be seen as a glutamate sink of infinite capacity. Instead noticeable amounts of the neurotransmitter will accumulate in the ECS and we need to keep track of the concentrations in the cleft and in the ECS. We also understand that cell swelling will play a major role.

#### Glutamate release

Glutamate is released in quanta of about 3,000 molecules and release depends on the membrane potential *V* in a threshold–like manner [67]. However, instead of modeling quantal release we propose a continuous release function. Glutamate release grows gradually (with a power law dependence) for depolarizations beyond a critical potential *V*_*cr*_. The model is set up such that for one action potential we get the expected release of 3,000 molecules per synapse. However, also smaller amounts can be released for more moderate depolarizations. While single synapses can indeed only receive glutamate in fixed quanta, this continuous release approximation should be seen in the context of the whole neuron. Neural processes are stochastic and also for depolarization events that are less pronounced than action potentials glutamate release in some synapses is expected. Our continuous release model approximates this behavior and the average glutamate release in a synapse is

$$J_{rel}\left(V\right)=\{\begin{array}{ll} R_{max}{\left(\frac{V-V_{cr}}{V_{hi}-V_{cr}}\right)}^{2}\frac{N_{i}^{G}}{N_{max}^{G}} & \text{for}\;V\geq V_{cr}\\ 0 & \text{for}\;V<V_{cr}. \end{array}$$ (1)

Note that we do not model the pre- and postsynaptic neurons individually and assume that both cells share the same microenvironment. This way, the single neuron can be considered both pre- and postsynaptic cell. This is equivalent to a network of identical units where each unit consists of one neuron, one astrocyte, and ECS. Each neuron experiences the same pre- and postsynaptic activity as other neurons in the network. While this simplified approach has been applied in other conditions [57, 58, 76], it is particularly a reasonable assumption in SD where the entire network is flooded with high concentrations of *K*^+^ and glutamate.

The parameters defining the glutamate release model are *V*_*cr*_ and high potential *V*_*hi*_ that defines the range of membrane potential where glutamate is released, and the maximal release rate, *R*_*max*_. We ignore spontaneous glutamate release by neuron, however, setting the critical potential *V*_*cr*_ to more negative value would allow such release. Adding such effect does not change our results significantly and is ignored.

Glutamate release also depends on the remaining glutamate, $N_{i}^{G}$, in the presynaptic terminals. We remark that it must be carried in vesicles to be released properly. Initially, the amount will be at the maximal level, $N_{max}^{G}$, but during SD it is reduced. Over the total duration of SD huge amounts of glutamate are released. Neurons and glia cells take it up from the cleft and the ECS, but at first the buffered glutamate is not enclosed in vesicles—it cannot be used for synaptic signals right away. Buffered glutamate gets recycled to produce new vesicles. The intracellular (IC) diffusion and recycling of the buffered glutamate into new vesicles slowly recover $N_{i}^{G}$ (see below).

**Fig 1:**
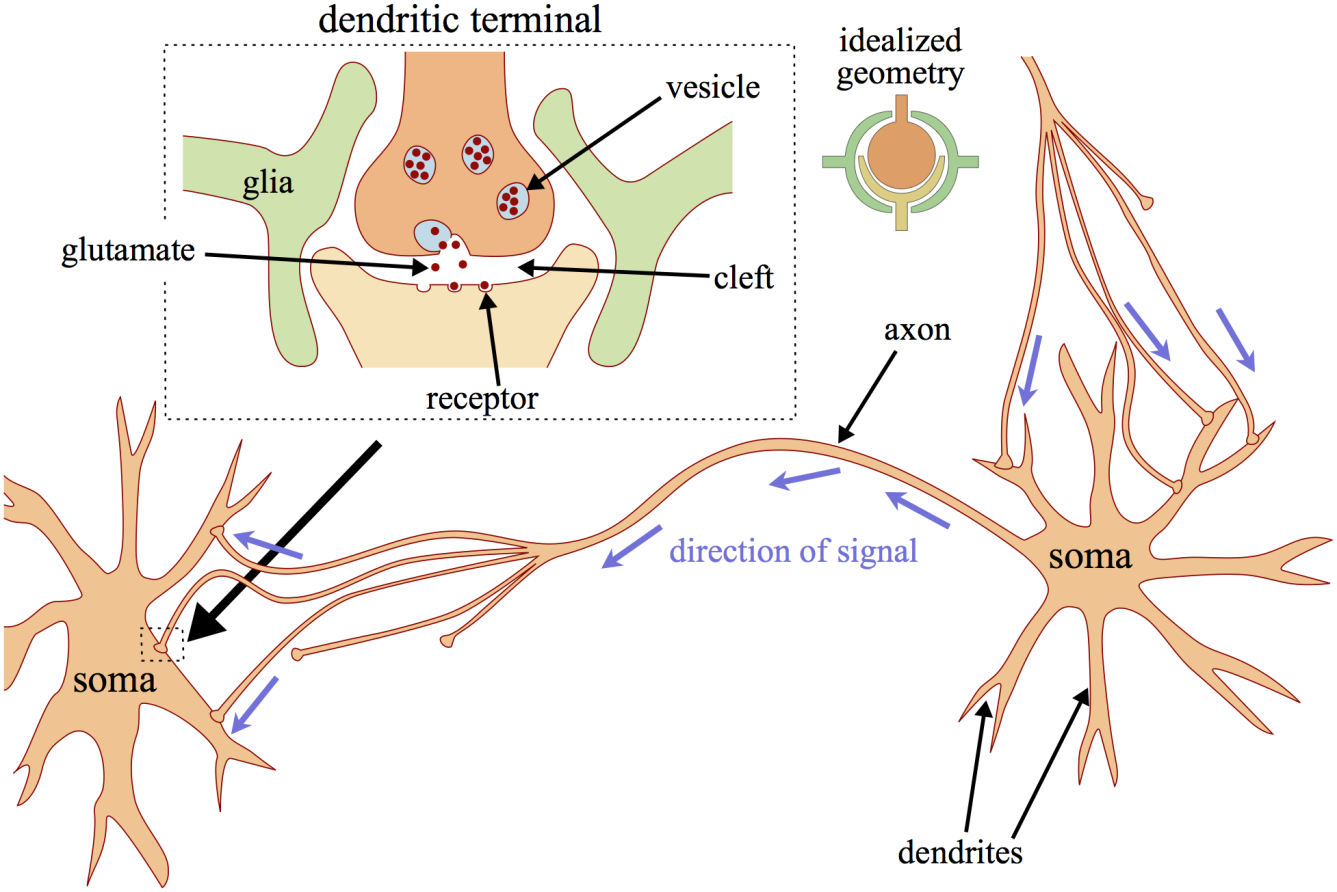
Neurons and synaptic connections. When a signal travels from the right to the left neuron along the axon, glutamate is released at the dendritic terminals. The terminals are separated from the neighboring neuron by the synaptic cleft. Signal transmission goes via release of the neurotransmitter glutamate. It binds to receptors on the postsynaptic neuron and can initiate new action potentials. The synaptic terminals are located at the dendrites of the receiving neuron. At the same time the right neuron can receive glutamate–mediated signals from other neurons through the dendritic terminals located near its own soma. The inset on the left shows additional details: glutamate is contained in vesicles and in the release process all molecules are released into the cleft at once. The inset on the right shows idealized geometry of the synapse considered in the model.

Glutamate is released into the synaptic cleft that is located at the dendritic terminal (see Fig 1). Its size is given by its height *h* and radius *r*. We assume a half–spherical shape and obtain the following cleft volume *ω*_*c*_ (Fig 1 inset):

$$\begin{array}{c} \omega_{c}=\frac{1}{2}[\frac{4}{3}\pi ({\left(r+h\right)}^{3}-r^{3})]\approx 2\pi r^{2}h \end{array}$$ (2)

Typical values for *r* and *h* are given in Table C in S1 Text [77, 57]. Terms of the order $O(h^{2})$ and higher are omitted, because *h* ≪ *r*.

Glia cells reach out to the dendritic cleft creating the so–called glial envelope [70] (Fig 1). We estimate the whole volume *ω*_*en*_ that is enclosed in this envelope to be three times as large as the cleft (Fig 1 inset):

$$\begin{array}{c} \omega_{en}=6\pi r^{2}h \end{array}$$ (3)

In the following, we will assume that glutamate in the cleft spreads into the whole envelope immediately after its release, i.e., the concentrations in the cleft and the envelope are the same and we refer to them synonymously. With this assumption we will only distinguish between glutamate concentrations in the ECS and the cleft and denote them by *G*_*e*_ and *G*_*c*_, respectively. The release of Δ*N*^*G*^ glutamate molecules leads to a cleft concentration of

$$\begin{array}{c} \frac{\Delta N^{G}}{\omega_{en}}=G_{c}\;. \end{array}$$ (4)

If we assume a baseline level of nearly zero, 3,000 molecules increase the concentration by 1.3 mM.

#### NMDA and AMPA receptor binding

Glutamate at high concentrations will bind to the receptors on the postsynaptic dendrites. Specifically it excites a neuron by binding to the NMDA or AMPA receptors. Computational models for the effect of receptor gates on action potentials have been developed for very different scenarios than SD. While SD involves all neural synapses, the computational models available are for normal action potential events involving only approximately $N_{syn}^{AP}=20$ synapses.

The effective receptor conductance in these events is in the range from 1e–8 to 6e–7 mS for NMDA, and from 3.5e–7 to 1e–6 mS for AMPA [71]. With a membrane surface area of 1.8e–4 cm^2^ and 5,000 synapses (note that we assume 50% of the 10,000 synapses to be pre-synaptic and the remaining 50% to be post-synaptic) that are activated at the same time, we obtain the following ranges for the maximal receptor conductance densities ${\bar{g}}_{NMDA/AMPA}$:

$$\begin{array}{c} \frac{0.3}{N_{syn}^{AP}}\;\text{mS}/{\text{cm}}^{2}\;<\;{\bar{g}}_{NMDA}\;<\;\frac{16.7}{N_{syn}^{AP}}\;\text{mS}/{\text{cm}}^{2}\;, \end{array}$$ (5)

$$\begin{array}{c} \frac{9.7}{N_{syn}^{AP}}\;\text{mS}/{\text{cm}}^{2}\;<\;{\bar{g}}_{AMPA}\;<\;\frac{27.8}{N_{syn}^{AP}}\;\text{mS}/{\text{cm}}^{2}\;. \end{array}$$ (6)

For example, considering the lower limit on NMDA receptors conductance of 1e–8 mS with $N_{syn}^{AP}=20$ leads to a conductance of 1e–8 mS/$N_{syn}^{AP}$ per synapse. Multiplying this number by 5,000 synapses and dividing by a membrane surface area of 1.8e–4 cm^2^ will result in the lower limit in Eq 5 ($1\times 10^{-8}\text{mS}\times 5,000/(N_{syn}^{AP}\times 1.8^{-4}{\text{cm}}^{2})$). For our simulations we assume action potential receptor conductances of 1e–7 mS and 3.5e–7 mS for NMDA and AMPA, respectively. This implies the SD–relevant conductances given in Table C in S1 Text.

NMDA and AMPA receptor gates open for Na^+^ and K^+^ ions, and the opening probability of the particular gate is described by the gating variables *r*_*NMDA*_ and *r*_*AMPA*_. Their dynamics are given by a Hodgkin–Huxley–like formalism with an additional dependence on the glutamate concentration *G*_*c*_ in the cleft [71]:

$$\frac{dr_{AMPA}}{dt}=G_{c}\alpha_{AMPA}\left(1-r_{AMPA}\right)-\beta_{AMPA}r_{AMPA}$$ (7)

$$\frac{dr_{NMDA}}{dt}=G_{c}\alpha_{NMDA}\left(1-r_{NMDA}\right)-\beta_{NMDA}r_{NMDA}$$ (8)

The parameters *α*_*NMDA/AMPA*_ and *β*_*NMDA/AMPA*_ were estimated by Destexhe et al. [71] by fitting both models to experimental data and are given in Table C in S1 Text. When compared to detailed Markov chain models with several gating states and taking into account the desensitization of the receptor, these simpler models were shown to fit the observed postsynaptic currents through NMDA and AMPA receptors equally well [71].

The receptor currents are given as

$$I_{Na/K}^{AMPA}={\bar{g}}_{AMPA}r_{AMPA}\left(V-E_{Na/K}\right)$$ (9)

$$I_{Na/K}^{NMDA}={\bar{g}}_{NMDA}r_{NMDA}\frac{V-E_{Na/K}}{1+0.33\left[{\text{Mg}}^{2+}\right]\exp\left(-0.07V-0.7\right)}$$ (10)

#### Diffusion and glutamate uptake

One important mechanism that clears glutamate from the cleft is diffusion. To estimate the glutamate diffusion rate we note that the cross section area, *A*_*σ*_, for fluxes from the envelope into the ECS is only 5% of the outer spherical surface of the dendritic connection, because 95% are covered by the glial envelope [70]:

$$\begin{array}{c} A_{\sigma }=0.05\cdot 4\pi r^{2} \end{array}$$ (11)

Let *D*_*G*_ be the glutamate diffusion coefficient [78] and Δ*x* the cutoff distance from the synapse at which the extracellular glutamate concentration is in a steady state [70]. Then we get the following flux of glutamate out of the cleft:

$$\begin{array}{c} J_{diff}=-A_{\sigma }\;\frac{D_{G}}{\Delta x}(G_{c}-G_{e}) \end{array}$$ (12)

Receptor binding is not a clearance mechanism and glutamate will be re–released into the cleft. What clears glutamate is uptake by the neuron and the glia cell. The mathematical description of cellular glutamate uptake is formulated in terms of the density of available binding sites on the neuron or glia cell *B*. As a chemical reaction scheme, glutamate uptake can be pictured as follows [70, 58]:

$$\begin{array}{c} G+B\underset{k_{-1}}{\overset{k_{+1}}{⇌}}GB\overset{k_{r}}{⇀}G^{up}+B \end{array}$$ (13)

We apply this scheme with different rates to model four uptake scenarios: uptake from the cleft or the ECS into the neuron or the glia cell. *G* is the glutamate concentration in the cleft or the ECS, and *B* is the neural or glial concentration of free binding sites through which the neurotransmitter can be transported into the cells. Bound glutamate is denoted by *GB*. It can either be re–released or taken into the cell. Both these processes leave a free binding site. Buffered glutamate is denoted by *G*^*up*^. Under the assumption that this reaction chain is stationary with a constant transporter concentration, *B*, the following uptake velocity, *v*, of glutamate into the cell, i.e. the velocity of the process *G* ⇀ *G^up^* can be derived (see [79] for this kind of derivation):

$$\begin{array}{c} v=\underset{v^{max}}{\underset{︸}{Bk_{r}}}\;\frac{G}{G+k_{m}}\;\text{with}\;k_{m}=\frac{k_{-1}+k_{r}}{k_{+1}} \end{array}$$ (14)

The velocity is measured in [mM/sec], and we note that in this phenomenological scheme the uptake ability is described by a spatial density *B* of binding sites. In a more detailed physiological description, glutamate uptake depends on the surface density *ρ*^*B*^ of binding sites in the cellular membrane. We assume that *B* is proportional to *ρ*^*B*^. Glia cells (subscript *g*) have about eight times more binding sites than neurons (subscript *n*) [80, 81, 82, 57].

$$\begin{array}{c} \rho_{g}^{B}=8\rho_{n}^{B} \end{array}$$ (15)

Moreover, uptake depends on the surface area *A*^*up*^ that is available for uptake. For glial uptake from the envelope we note that glia cells cover the synapse from the outside which is approximately one spherical surface area (see Fig 1). Neural uptake is through those parts of the neural membranes facing this glial envelope (one spherical surface), and from below and above the cleft (two half–spherical surfaces) which makes the neural uptake area twice as large:

$$A_{c\to n}^{up}=8\pi r^{2}$$ (16)

$$A_{c\to g}^{up}=4\pi r^{2}$$ (17)

Assuming that uptake depends on these surface areas in a proportional fashion as well, we conclude that

$$\begin{array}{c} v_{c\to g}^{max}=4v_{c\to n}^{max}\;, \end{array}$$ (18)

when we apply Eq (14) to neural and glial glutamate clearance from the cleft.

To extend this scheme and model uptake from the ECS, we need to take the role of volume into account. If the volume in which the glutamate is present was twice as large, reducing the concentration by a certain amount would take twice as long. So there is an inverse relation between volume and uptake velocity since the latter relates to changes in concentration. In summary, we assume the following dependencies

$$\begin{array}{c} v^{max}\;\propto \;\frac{\rho^{B}A^{up}}{\omega }\;. \end{array}$$ (19)

The transporter densities $\rho_{n/g}^{B}$ are taken to be constant throughout the cellular membranes and the contact surfaces for uptake from the ECS are simply the whole membrane areas from Table B in S1 Text:

$$A_{e\to n}^{up}=A_{m}^{\left(n\right)}$$ (20)

$$A_{e\to g}^{up}=A_{m}^{\left(g\right)}$$ (21)

These assumptions imply the following relation of uptake velocities:

$$\begin{array}{c} \frac{v_{c\to n/g}^{max}}{v_{e\to n/g}^{max}}=\frac{A_{c\to n/g}^{up}}{\omega_{en}}\;\frac{\omega_{e}}{A_{e\to n/g}^{up}} \end{array}$$ (22)

With the volumes and areas from Eqs (3), (16), (20), (21) and Table B in S1 Text this yields

$$v_{e\to n}^{max}=0.12\;v_{c\to n}^{max}$$ (23)

$$v_{e\to g}^{max}=0.24\;v_{c\to g}^{max}$$ (24)

These coefficients are computed using the resting volume $\omega_{e}^{0}$. Taking into account volume changes we get

$$v_{e\to n}^{max}=0.12\;v_{c\to n}^{max}\;\frac{\omega_{e}^{0}}{\omega_{e}}\;,$$ (25)

$$v_{e\to g}^{max}=0.24\;v_{c\to g}^{max}\;\frac{\omega_{e}^{0}}{\omega_{e}}\;.$$ (26)

The volume of the envelope *ω*_*en*_ is constant. In our model we will only vary $v_{c\to e}^{max}$, the other uptake velocities are then implied by Eqs (18), (25) and (26). Altogether glutamate dynamics are described by four dynamical variables: the average amount of IC glutamate near the synapse $N_{i}^{G}$, the average amount in the cleft $N_{c}^{G}$, the total amount in the ECS $N_{e}^{G}$, and buffered glutamate $N_{up}^{G}$ that has not been recycled. The rate equations are

$$\frac{dN_{i}^{G}}{dt}=-J_{rel}+k_{rec}N_{up}^{G}$$ (27)

$$\frac{dN_{c}^{G}}{dt}=J_{rel}+J_{diff}-\omega_{en}\left(v_{c\to n}+v_{c\to g}\right)$$ (28)

$$\frac{dN_{e}^{G}}{dt}=-\frac{N_{syn}}{2}J_{diff}-\omega_{e}\left(v_{e\to n}+v_{e\to g}\right)$$ (29)

$$\begin{array}{lll} \frac{\text{d}N_{up}^{G}}{\text{d}t} & = & \frac{N_{syn}}{2}\omega_{en}\left(v_{c\to n}+v_{c\to g}\right)\\ & & +\omega_{e}\left(v_{e\to n}+v_{e\to g}\right)-k_{rec}N_{up}^{G} \end{array}$$ (30)

Note the factor *N*_*syn*_/2 in Eq (29): only 50% of glutamate that leaves the synaptic clefts goes into the ECS associated with our local average neuron.

Uptake of glutamate goes along with ion cotransport [66]. Thus the rate equations for membrane potential (Eq. 1S) and ion dynamics (Eq. 17S–19S) change according to Eqs. (35S–37S, S1 Text).

#### Numerical methods

The model equations were implemented in the widely available numerical continuation package AUTO [83]. To facilitate the dissemination of these results, the AUTO code reproducing the key results in this paper is given in S2 Text and also archived at modelDB [84].

### Experimental methods

#### Animals

Experiments were approved by University of Houston Institutional Animal Care and Use Committee. Studies were performed on juvenile, 15-24 days old, male wild type Sprague Dawley rats. K^+^-induced SD is well documented to form in the second postnatal week and is well developed by P15 [85, 86, 87]. We used 7 animals for the TFB-TBOA group, and 9 animals for control. Number (n) in the paper refers to number of slices used in the experiments.

#### Solutions and drugs

For tissue dissection and slice preparation oxygenated (95% O2, 5% CO2) high sucrose dissection buffer (in mM): 248.3 sucrose, 2.5 KCl, 1.25 NaH2PO4, 3 MgSO4, 10 MgCl2, 0.5 CaCl2, 26 NaHCO3, and 10 dextrose) was used. Slices were pre-incubated in aerated (95% O2-5% CO2) standard artificial cerebrospinal fluid (ACSF) containing (in mM) 130 NaCl, 1.2 MgSO4, 3.5 KCl, 1.2 CaCl2, 10 glucose, 2.5 NaH2PO4, and 24 NaHCO3 (pH7.3). For induction of SD, high K^+^ solution was made by an equimolar replacement of NaCl with 26 mM KCl. 2% Dimethyl sulfoxide (DMSO) and 98% deionized water solution was used to dissolve TFB-TBOA powder (Tocris Bioscience). TFB-TBOA was used with the final concentration of 50 nM, IC50 value of 17 nM and 22 nM for GLT-1 and GLAST, respectively [88].

#### Visual cortex slices preparation

Standard in vitro electrophysiology slice preparation techniques were used. In brief, the rats were deeply anesthetized with ether and decapitated using a guillotine. After the decapitation, the brain was removed rapidly and placed in ice-cold high sucrose dissection solution. After isolation of visual and somatosensory cortex, coronal slices (350 *μ*m) were prepared using a vibratome (Technical Products International)), transferred to a room temperature incubation chamber and warmed to 30°C [89]. Following the incubation period, individual slices were transferred to a recording chamber with oxygenated ACSF perfusing constantly at a rate of 2-3 ml/min at 30°C [89].

#### Electrophysiology

To record extracellularly, recording pipettes were made from borosilicate glass capillaries using Flaming/Brown model P-97 horizontal micropipette puller (Sutter Instruments CO.). The extracellular (EC) recording electrodes were filled with 0.9% NaCl (1-2 MΩ). Electrodes were placed in layers 2-3 of visual cortical slices. Control ACSF was replaced with high KCl (26mM) ACSF and warmed to 36C. Multi-Clamp Commander (MCC) 700 amplifiers (Axon Instruments) were used for all electrical recordings. Data were low-pass filtered and digitized at 1 KHz for extracellular and 10 KHz for whole-cell recordings (Digidata; pCLAMP,Molecular Devices).

SD in individual pyramidal neurons of layers 2-3 of visual cortex were recorded using whole-cell patch clamp technique. Borosilicate micropipettes (4-9MΩ) contained IC solution (in mM): 116 K-gluconate, 6 KCl, 0.5 EGTA, 20 HEPES, 10 phosphocreatine, 0.3 NaGTP, 2 NaCl, 4 MgATP; adjusted to pH 7.25 and osmolarity of 295 mOsm. After the initiation of SD, high KCl solution was turned off and replaced with control ACSF. SD was defined as the beginning of rapid depolarization in individual neurons and network to the time when the membrane potential repolarized to its pre-SD value.

Slow ramp depolarizations (10-40 mV/1000ms) from the cell resting membrane potential were used to determine action potential threshold and cell spiking properties. Incremental hyperpolarizing and depolarizing current injections were used to study the passive and active neuronal membrane properties (10 pA increments for 500-1000 ms) [89]. More details about experimental methods can be found in [90].

#### Data and statistical analysis

Graph pad Prism 5 software was used for analyzing the data. All data are expressed in mean ± SEM. Unpaired student’s t-test with Welch’s correction was used to determine significant differences between the two groups. The value of p<0.05 was regarded as statistically significant.

## Results

The above introduced model allows us to study glutamate and ion dynamics simultaneously. The glutamate model as such relies on a number of simplifying assumptions like the continuous release function we introduced in Eq (1) or using the same uptake mechanism for glia cells and neurons. Other key factors controlling glutamate homeostasis and glutamate-dependent fluxes such as the influx of calcium ions through NMDA receptors, the detailed gating kinetics of NMDA and AMPA receptors, desensitization of AMPA receptors, the reversal of glutamate transport under high EC K^+^ and IC glutamate, extra-synaptic receptors, and the dependence of glutamate-glutamine cycle on ATP are not included in this model. Furthermore, the release of glutamate induced by the gain of function of presynaptic Ca^2+^ channel, Cav2.1, in familial hemiplegic migraine (FHM) type 1 are also not included in our model. Our focus in this study is to investigate how glutamate influences ion dynamics rather than interpreting the details of the glutamate release and uptake itself. Nevertheless, connecting the ion dynamics with glutamate necessitates the minimal details included in our model and cannot be studied using simple release and exponential decay of glutamate. Most importantly, we analyze how glutamate clearance, particularly through glutamate transporters, affects ion dynamics during SD and the duration and termination of SD. Results are obtained from direct numerical simulations as well as bifurcation and phase space analyses.

### Spreading depolarization with intact glutamate clearance

**Fig 2:**
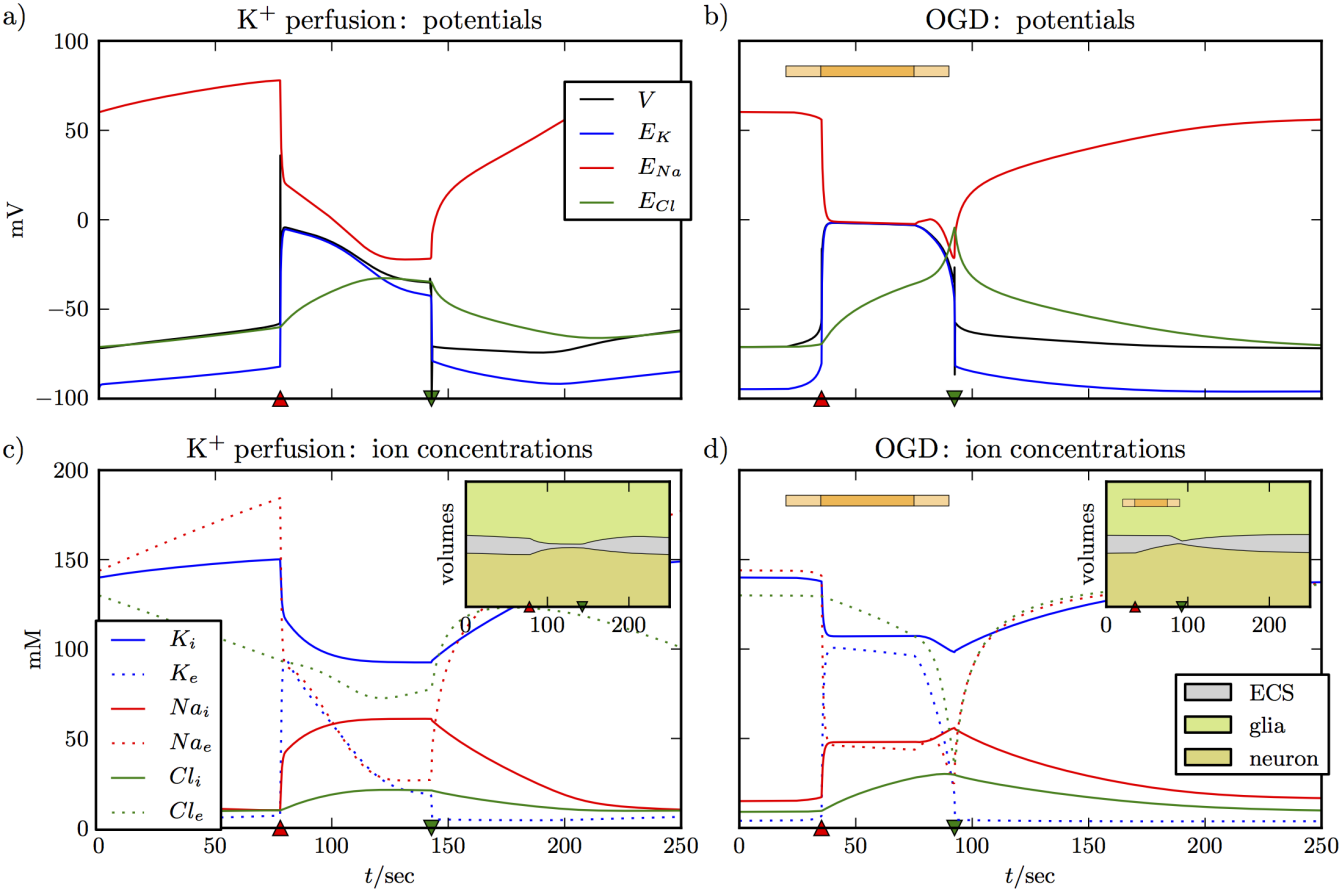
Two variants of SD with intact glutamate clearance. The left panels **(a)** and **(c)** show SD induced by perfusion with high K^+^, the right panels **(b)** and **(d)** are for OGD. The OGD protocol is indicated by the horizontal bar where the light sections at the beginning and the end indicate the smooth de– and reactivation of the regulatory functions. The figure shows all the familiar aspects of SD. In **(a)** and **(b)** the neuron depolarizes and the differences between Nernst potentials become very small. Depolarization is maintained for about 70 sec and ends with an abrupt repolarization drop after which the Nernst potentials slowly return to their initial values. Changes in ion concentrations in **(c)** and **(d)** correspond to the evolution of the potentials. There is, for example, a huge increase in extracellular K^+^ and a huge drop in extracellular Na^+^. The ion fluxes induce swelling of the glia cell and the neuron (see the insets), which result in shrinkage of the ECS. The time of de– and repolarization are marked by a red upward pointing and a green downward pointing triangle. These symbols will be used in the following figures to indicate these events.

We look at SD caused by perfusion of brain slices with high K^+^ and SD caused by OGD. The first case is modeled by increasing *K*_*bath*_ (K^+^ in the bath) from 4 mM to 15 mM at the start of simulation and stays elevated throughout the experiment. For the second situation all pump and glia functions slowly cease within 15 sec, remain interrupted for 40 sec, and are then slowly reactivated within 15 sec. Fig 2 shows the evolution of the membrane potential, Nernst potentials, ion concentrations, and the volumes. The OGD protocol is indicated by the orange bar in Fig 2b and 2d. The light parts at the beginning and end of the bar mark the smooth cessation and reactivation, respectively. In OGD, the uptake parameter $v_{c\to n}^{max}$ is also slowly set to zero and glutamate clearance is interrupted as well. The normal value of $v_{c\to n}^{max}$ is 0.03 mM/msec. This is in the range of values suggested by Rusakov, and Slichenko and Tass in Refs. [70, 58].

The main result of Fig 2 is that our addition of glutamate–related processes does not change the familiar course of events of SD. In both cases SD begins with a short burst of spikes driving the neuron into depolarization block. The burst causes huge changes in ion concentrations, most prominently a drop in extracellular Na^+^ and a huge rise in extracellular K^+^ (see curves for *Na*_*e*_ and *K*_*e*_ in Fig 2c and 2d). The extracellular space is also rapidly flooded with high concentration of glutamate that prohibits the ion channels from closing. As a consequence of these ion changes, the membrane potential differences become very small (see Fig 2c and 2d) and the neuron enters a phase of sustained depolarization very quickly. The time at which depolarization begins is marked by a red triangle pointing upwards. Thereafter the neuron remains depolarized for about 60 sec before it suddenly repolarizes and ion concentration begin to recover. Note that this abrupt transition leads to a brief overshoot into hyperpolarization. The time of this potential drop is marked by a green triangle pointing downwards.

The main difference between SD caused by *K*^+^ perfusion and OGD in the model is that in the perfusion experiments extracellular K^+^ (and other concentrations because of it) builds slowly as K^+^ diffuses from bath to ECS till it reaches a point where the cell starts spiking and enters SD. The cell comes out of SD spontaneously as the *K*^+^ clearance mechanism overcomes the release processes. If the simulation is allowed to run for longer time, the cell repeats this process till *K*_*bath*_ is reduced back to physiological values. Another difference between SD caused by K^+^ perfusion and OGD is that in the latter case there is no spiking in the beginning of SD.

The insets of Fig 2c and 2d show how the volumes of the neuron, the glia cell, and the ECS change during these ion fluxes. The ECS shrinks dramatically while the neuron is depolarized. During OGD, ECS shrinkage gets much faster when ion pumps and glial functions are slowly reactivated. The reason is that glia swelling is blocked during OGD since there is no particle uptake. After reactivation of regulatory functions, the glia cell starts swelling because of K^+^ uptake and ECS shrinkage is accelerated immediately.

**Fig 3:**
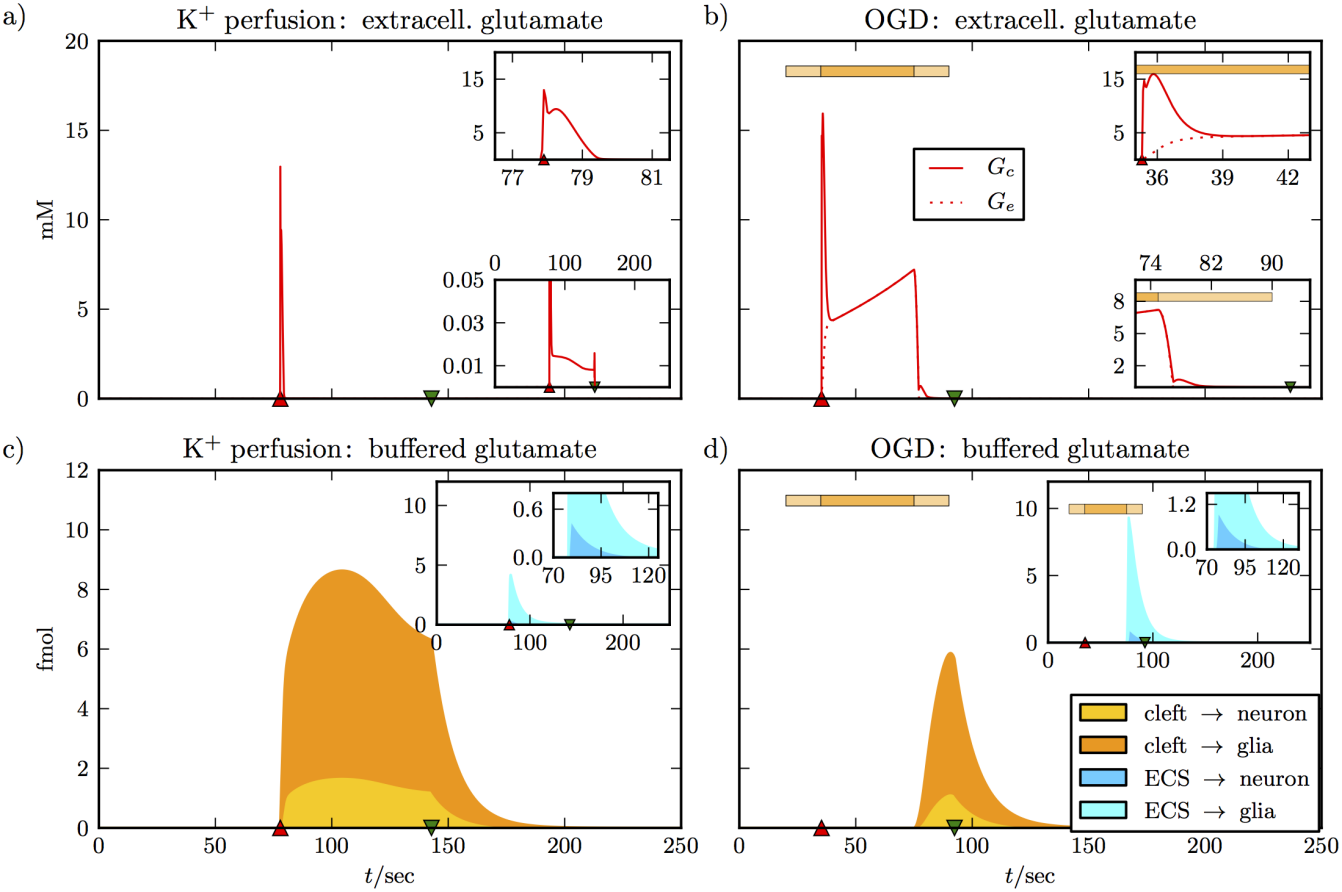
Glutamate dynamics for the simulations from Fig 2. Panels **(a)** and **(b)** show a jump of the cleft glutamate concentration to about 15 mM when the neuron depolarizes. In the simulation of K^+^ perfusion we assume intact glutamate uptake at all times, and the high cleft concentration is brought back to a much lower level within 2 sec (see upper inset of **(a)**). There is a plateau concentration near 0.01 mM that is maintained as long as the neuron is depolarized, and is only cleared after repolarization (lower inset of **(a)**). There is no noticeable glutamate elevation in the ECS. In panel **(b)** we see different dynamics, because there is no glutamate clearance during OGD. The sharp jump in the cleft concentration also decays within 2 sec, but the concentration then settles to a much higher level of 5 mM (see upper inset). It keeps increasing slowly until glutamate clearance is slowly reactivated (see horizontal bar for the OGD protocol). The lower inset shows that glutamate is back to a very low level before the neuron repolarizes. The extracellular concentration goes up to more than 5 mM. When there is no glutamate clearance at all (center part of the OGD bar) the concentrations in the cleft and the ECS are equal because of diffusion. The lower panels show pathways of glutamate clearance. Glutamate that has been taken up from the cleft or the ECS by either the neuron or the glial cell, but has not yet been recycled, counts as buffered glutamate (see Eq 30). The main plots show glutamate clearance from the cleft, the inset show clearance from the ECS. For K^+^ perfusion, more glutamate is cleared directly from the cleft than from the ECS (see peak values in the main plot and inset of panel **(c)**). In the cleft, more glutamate is cleared by glia than by the neuron (compare the orange and the yellow portion of the total uptake from the cleft). In the ECS, this relation is even more pronounced (compare turquoise to blue in the second inset). In panel **(d)**, glutamate clearance only sets in with the reactivation of regulatory functions at the end of the OGD protocol. Now more glutamate is cleared from the ECS, since there is more in the ECS than in the clefts. The relation between uptake by glia and neural uptake is consistent with **(c)** and Eqs (26) and (18).

The potential and ion dynamics for high K^+^ perfusion and OGD are very similar. However, as Fig 3 shows the glutamate dynamics clearly differ. Fig 3a and 3b show extracellular glutamate concentrations in the synaptic cleft and in the ECS. De– and repolarization times are indicated by triangles again. We note that depolarization increases the glutamate concentration in the cleft to more than 10 mM in both cases. The insets of Fig 3a and 3b show more details on finer scales. The sharp peak of the concentration in the cleft decays within 2 to 3 sec (upper insets). For OGD, this goes along with a rise in glutamate in the ECS due to diffusion. The two concentrations are equal after 3 sec. For K^+^ perfusion, *G*_*e*_ remains practically zero at all times. In the lower inset of Fig 3a, we see that after the peak *G*_*c*_ goes to values between 10 and 20 *μ*M, while in the OGD simulation, the level remains 5 mM in the cleft and the ECS. Concentrations go back to zero with repolarization. The lower inset of Fig 3b shows that the neurotransmitter is cleared quickly when neuronal and glial uptake functions are reactivated. Recall that both are impaired during OGD. Concentrations are reduced to values in the range of 10 to 20 *μ*M within a few seconds before repolarization takes them to zero.

Fig 3c and 3d show the different uptake pathways. Main plots present uptake from all of the synaptic clefts of the neuron. The total height of the colored region is total uptake, the yellow and the orange portions are the specific contributions of the neuron and the glia cell. By buffered glutamate we mean molecules that have been taken up by the cells, but have not been recycled. Vesicle reproduction reduces $N_{up}^{G}$, which is why the amounts of buffered glutamate do not strictly grow, but can also shrink (see Eq (30)). In the OGD run, uptake dynamics only start with uptake reactivation by the end of OGD, so the different time signature is simply dictated by the OGD protocol.

What these uptake plots show us is that glial uptake is dominant for clearance from both the clefts and the ECS (see insets). Moreover, more glutamate is cleared from the ECS than from the cleft in OGD–caused SD. The reason is that the amount of glutamate that diffuses into the ECS is larger than the amount that remains in the clefts. Also in perfusion–caused SD, large amounts of the neurotransmitter are taken up from the ECS as well. This type of uptake is fast enough to maintain a concentration *G*_*e*_ that is nearly zero at all times. We like to stress that Fig 3 should only be seen as an overview of the glutamate dynamics that our model provides. Details like the concentration plateau that follows the depolarization peak depend on the fine balance of release, uptake, and glutamate recycling. A different release function and a more detailed incorporation of the recycling process could lead to a different plateau. The most reliable aspects of our model are the amount of glutamate release during the depolarization burst, the diffusion process, and cellular glutamate uptake, since these parts of the model have been developed based on experimental data [58, 70, 67].

### Impaired glutamate clearance delays recovery

As pointed out above, increasing the expression of astrocytic glutamate transporters has been shown to reduce the infarct volumes following ischemia [32] and protects against the onset of ischemia-induced SD [33]. A recent study shows reduced rates of glutamate and K^+^ clearance by cortical astrocytes during neural activity and reduced density of excitatory amino acid transporters 1a (EAAT-1a) in cortical perisynaptic astrocytes in heterozygous FHM type 2-knockin mice [91]. By partial inhibition of glutamate transporters in wild-type mice, this study provides clear evidence that defective glutamate clearance can account for most of the facilitation of SD initiation in FHM type 2-knockin mice [91]. *In vitro* studies showed impaired glutamate uptake in hippocampal mixed astrocyte-neuron cultures from mice expressing FHM type 2-causing *α*_2_ Na^+^/K^+^ ATPase. Induction of SD in these animals resulted in reduced recovery [92].

**Fig 4:**
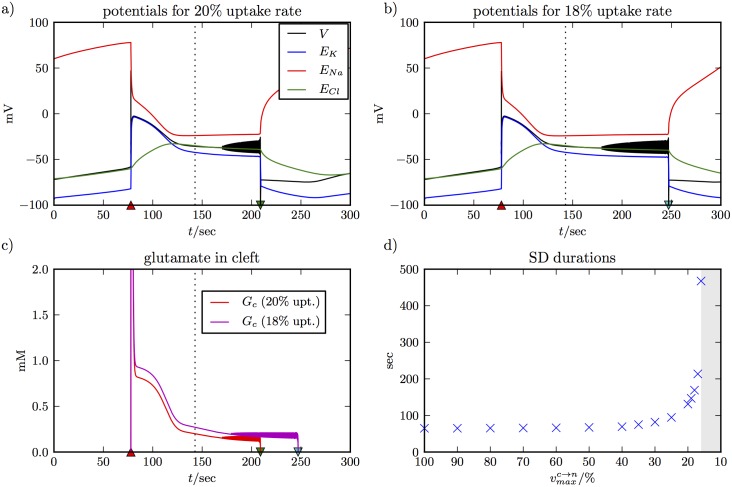
SD simulations with reduced uptake rates. Panel **(a)** and **(b)** show SD for K^+^ perfusion with uptake rates reduced to 20% and 18% of the normal value from Table C in S1 Text. The plots show that repolarization and recovery are delayed and the delay is longer for more reduced glutamate uptake. The repolarization time with normal uptake is indicated by the vertical dashed line. Before repolarization, the system shows low amplitude membrane potential oscillations. Panel **(c)** shows the evolution of the glutamate concentration in the synaptic clefts during the two simulations. Delayed recovery correlates with slower glutamate clearance. Before repolarization, there are glutamate spikes because of the membrane potential oscillations. Panel **(d)** gives an overview of SD durations for reduced uptake rates. In our model, the effect becomes noticeable only for rates of 35% and less. Extreme durations can be up to 500 sec, and recovery fails for uptake rates of less than 16%.

In the following, we provide a complete understanding of the glutamate uptake processes and its role in SD by reducing $v_{c\to n}^{max}$ (rates for other uptake pathways are implied). The simulations are shown in Fig 4 and they are consistent with our experimental observations (see below). Fig 4a and 4b show the membrane potential dynamics for maximal uptake rates of 20% and 18% of the $v_{c\to n}^{max}$–value used in Figs 2 and 3. The repolarization time for normal uptake is about 143 sec as indicated by the vertical dashed line. With the lower uptake rates, recovery is delayed by about 65 and 105 sec, respectively. Before repolarizing, the membrane potential oscillates with a low amplitude.

We note that the potentials for the first 143 sec into SD are the same in Figs 2a and 4a and 4b. This is also true for the ion concentrations, and we conclude that for the first 143 sec the ion fluxes are almost not affected by the smaller *v*^*max*^–values. This implies that the contribution of the cotransport currents $I_{ion}^{co}$ from Eq. (32S)–(34S) is negligible. The only difference for the first 143 sec between normal and impaired uptake lies in the much higher glutamate concentrations *G*_*c*_ (see Fig 4c vs the lower inset of Fig 3a). In other words, too much glutamate in the cleft prevents recovery. The neuron only repolarizes when the concentration is small enough. An uptake rate of 20% achieves this sooner than the one at 18%. In Fig 4d, we look at this delaying effect systematically and compare SD durations for a range of uptake rates between 100% (normal value) and 16%. The effect becomes noticeable at 35% and lower with a maximal duration of almost 500 sec for 16% uptake. Below 16% recovery fails. For uptake rates between 100% and 50% the duration of SD is nearly constant. In this range of uptake rates the evolution of *G*_*c*_ is comparable to Fig 3a. The initial jump in *G*_*c*_ is quickly reversed and recovery is unaffected by glutamate. Only for uptake rates of 35% and less do we see a slow down–regulation of *G*_*c*_ as in Fig 4c. We expect critical uptake rates to depend very sensitively on all processes of the glutamate cycle. The numerical values in Fig 4d are likely to differ between models and should not be assumed for a real system. However, we believe that the basic correlation between the duration of SD and glutamate regulation is a real effect that explains the delayed recovery from perfusion-induced SD in tissues that are exposed to TBOA in our experiments (see below). A recent study showed that 0.5 and 1 mM TBOA prolonged SD by 148% and 426% respectively [28]. A respective increase of 167% and 374% in glutamate concentration was observed in the same experiments.

### How glutamate affects the phase space

We can understand delayed recovery and recovery failure in SD from a phase space perspective. Let us briefly review the general method for the case of intact glutamate uptake (see Figs 2 and 3). The role of glutamate clearance is addressed in a second step. One can determine the timescales of the different processes in the model by a dimensionality analysis, and it turns out that $\Delta N_{bath}^{K}$ and $\Delta N_{glia}^{K}$ are the slowest variables (see Ref. [46] for a complete timescale analysis of a very similar model). Please note that in our model presentation it is not possible to read off the timescales from the coupling parameters. They have different units and consequently different orders of magnitude. A dimensionless presentation can be obtained by appropriate rescaling of the dynamical variables, but this is not our focus here. The timescales of vascular coupling and glial buffering have been derived rigorously in the above mentioned study [46]. These processes are the slowest ones in the system and they occur on similar timescales. This can be used to apply a so–called slow–fast analysis which allows us to derive the threshold condition for repolarization. Let us combine the two quantities to define a single slow variable:

$$\begin{array}{c} \Delta N^{K}≔\Delta N_{glia}^{K}+\Delta N_{bath}^{K} \end{array}$$ (31)

In a slow–fast analysis, we ask which states of the system are possible when a certain value of the slowest variable is given. To find these states we treat this variable as a parameter, which formally defines a subsystem of only the fast dynamics. The processes of the fast subsystem are membrane dynamics and ion fluxes across the neural membrane. We refer to them as transmembrane dynamics. For the fast subsystem we also treat glutamate as a parameter. This is not an approximation, because glutamate is fast. However, it is the only way to systematically study how elevated glutamate levels affect the other processes. For now we set the level extremely low (0.0001 mM), which practically means we ignore glutamate. This assumption is good enough at the de– and repolarization points.

Depending on our choice of Δ*N*^*K*^, the fast subsystem has a certain number of stable and unstable fixed points. With the help of the software tool AUTO [83], these fixed points can be found and followed, while Δ*N*^*K*^ is varied within a certain range. Every stable fixed point of the ‘fast subsystem’ is stable in the full system except for dynamics of Δ*N*^*K*^. When the timescale separation is sufficiently large the fast variables equilibrate to one of these stable fixed points, and this way the dynamics of the whole system will be guided by the fixed point structure of the subsystem. Formally, this concept is known as a ‘quasi–steady–state reduction’ [93].

**Fig 5:**
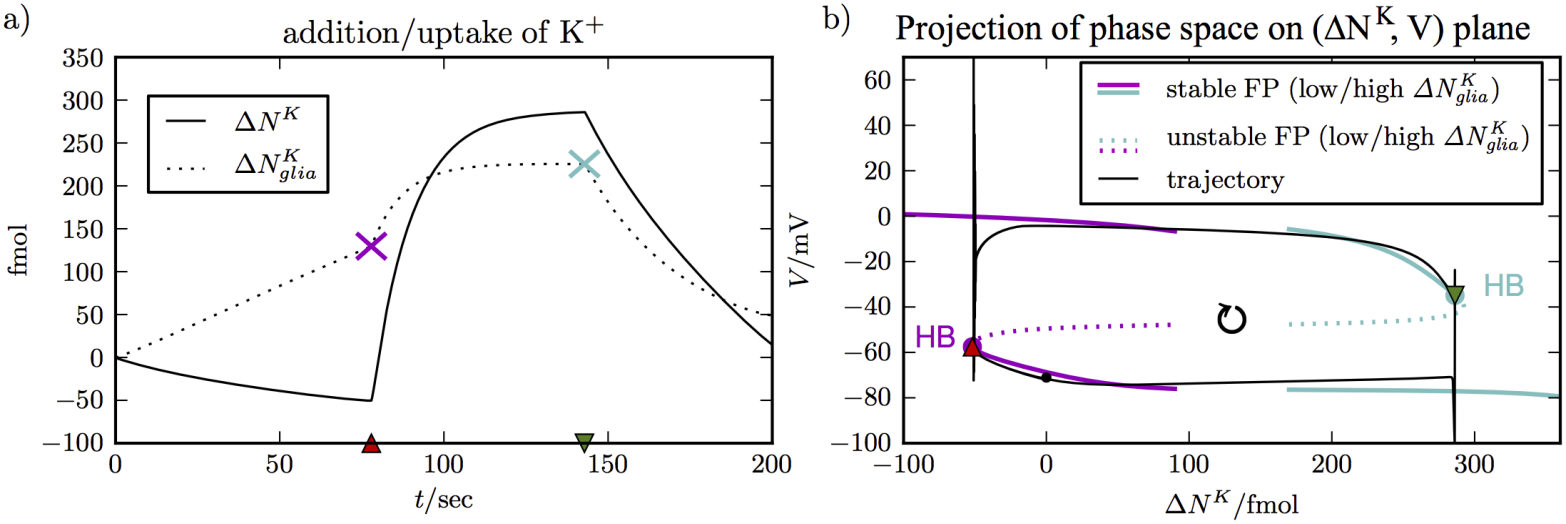
Phase space perspective of SD. Panel **(a)** shows the evolution of slow variables. Δ*N*^*K*^ takes extremum values at de– and repolarization. It is used as a bifurcation parameter to derive the fixed point structures in panel **(b)** that guide the trajectory in phase space. $\Delta N_{g}^{K}$ contributes to Δ*N*^*K*^ and its evolution is also shown. The values at the two points of interest are indicated by colored X markers. The values are used to compute the fixed point structures that guide the dynamics near the de– and repolarization point in **(b)**, respectively.

In Fig 5, we see how this works. The left panel (Fig 5a) shows the evolution of Δ*N*^*K*^ over 200 sec. It is indeed slower than the other variables (see Fig 2) with the exception of *Cl*_*i*/*e*_—a detail that has been addressed previously and can be neglected here [56]. Note that $\Delta N_{glia}^{K}$ and $\Delta N_{bath}^{K}$ are two independent dynamical variables, they affect volumes differently. So knowing Δ*N*^*K*^ alone is not enough information and we need to specify $\Delta N_{glia}^{K}$ too. That is why, $\Delta N_{glia}^{K}$ is also included in Fig 5a. We have marked the crucial values at the de– and repolarization points by a pink and a turquoise X respectively. We see that the neuron depolarizes at the minimal value and repolarizes at the maximal value of Δ*N*^*K*^. This means that SD starts when the K^+^ content of the neuron and its ECS is elevated, and that recovery is on the other hand only possible when enough K^+^ is taken away (recall the sign of the difference terms in Eq. (22S)).

The relation between Δ*N*^*K*^ and these events becomes more clear in Fig 5b, which shows the location of the fixed points of the fast subsystem in the (Δ*N*^*K*^, *V*)–plane and how the trajectory of the full system is guided by them. Near the depolarization point, we use the fixed point curves for $\Delta N_{glia}^{K}\approx 129\;\text{fmol}$ and near the repolarization point we use $\Delta N_{glia}^{K}\approx 226\;\text{fmol}$. Note that we do not show the complete fixed point curves. The entire curves are both z–shaped and overlap strongly. Hence for clarity, we only show two disconnected portions of these curves. The trajectory of the full system is a closed loop in the phase space and the transition points can now be understood through the fixed point structure.

Let us focus on the repolarization process. Depolarization can be explained analogously, and we refer the reader to a previous study for more details on both transitions [46]. As the trajectory approaches the repolarization point, it is closely guided by the stable depolarized fixed point curve. The potassium content decreases until the curve becomes unstable. Stability changes of fixed points generically occur in Hopf bifurcations or limit point bifurcations. The AUTO software provides this information and shows that the above change of stability is due to a Hopf bifurcation. For completeness, we mention that around a Hopf bifurcation there are always stable or unstable limit cycles. These limit cycles imply oscillation, which we often see shortly before repolarization. Typically, stable limit cycles in SD models only exist in a narrow range of Δ*N*^*K*^ values around the Hopf bifurcation before they change stability in a limit point bifurcation of limit cycles (see Figs 2 and 3 in Ref. [46] where ${\tilde{K}}_{e}=-\Delta N^{K}/\omega_{e}$).

When there is no stable upper fixed point or limit cycle the trajectory drops back onto the polarized stable fixed point branch. Since the transition from depolarization to repolarization happens at or very close to the Hopf bifurcation, the two can be related to each other. We remark that the situation will be different when the upper and lower fixed point branches do not overlap (see below). The tracking of limit cycles in our model is numerically very involved, because of the many different timescales. The continuation of limit cycles is hence beyond the scope of this study, but a complete analysis for a similar model has been performed before [46]. Note that our bifurcation diagrams only contain the bifurcations that change the stability of the fully stable fixed points. Subsequent bifurcations that change the degree of instability are not relevant to our analysis here and are omitted.

From the phase space perspective, we understand that recovery from SD relies on the existence of a stable repolarized state in the fast subsystem. In Fig 5b, such a state is available and also seems to exist with higher glutamate concentrations as in Fig 4a and 4b.

We now increase *G*_*c*_ and see how the fixed point curve changes. Unlike the treatment of Δ*N*^*K*^ as a parameter, this is not an approximation in the sense of a slow–fast analysis. Glutamate–related processes happen on fast timescales (see Fig 3a and 3b). We rather treat *G*_*c*_ as a parameter to study the effect of glutamate in some extreme scenarios. For example, how does high glutamate concentration in the cleft—that may occur with impaired clearance—affect the dynamics of the system? By fixing *G*_*c*_, we can obtain qualitative insights and answers to such questions.

**Fig 6:**
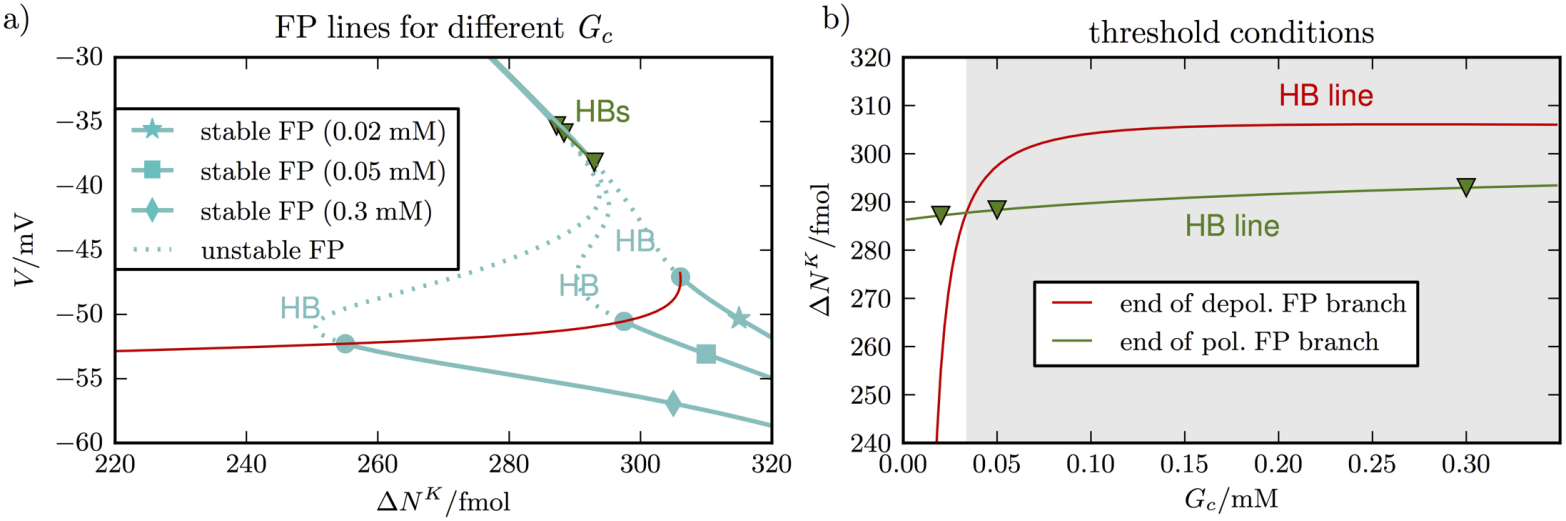
Dependence of the fixed point structure on *G*_*c*_. Panel **(a)** shows the fixed point curves for three different *G*_*c*_–values. The beginning of the polarized fixed point branch is shifted towards larger Δ*N*^*K*^–vaues as *G*_*c*_ increases. The depolarized and polarized branch only overlap for *G*_*c*_ = 0.02 mM. Scanning through all values of *G*_*c*_ from 0 to 0.3 mM and higher yields the red and green line indicating the beginning of the lower and the end of the upper stable fixed point branches. The green line is only a short curve connecting the triangle markers indicating the ends of the three upper fixed point branches. In panel **(b)** these curves are shown in the (*G*_*c*_, Δ*N*^*K*^)–plane. As long as the lower branch begins before the upper branch ends, i.e. whenever the green curve is above the red curve, repolarization is possible. The critical value of intersection is near 0.032 mM and for higher values of *G*_*c*_ there is no recovery (shaded region).

To discuss recovery, we look at the fixed point curves for high $\Delta N_{glia}^{K}$. Fig 6a shows these curves for three different values of *G*_*c*_. The two lower values 0.02 mM and 0.05 mM are chosen to show that the fixed point structure is very sensitive to *G*_*c*_. The highest value 0.3 mM is included because it is near the glutamate levels that prevent early recovery in Fig 4c (see values at the dashed vertical line).

The fixed point curves have a stable depolarized (upper) branch that ends at a maximal value of Δ*N*^*K*^ and a stable polarized (lower) branch that begins at a minimal value of Δ*N*^*K*^. These points are defined by Hopf bifurcations. The red line contains all the lower branch Hopf bifurcations for *G*_*c*_–values between 0 mM and 0.35 mM, the green line contains the upper branch Hopf bifurcations. The polarized branch shifts towards higher Δ*N*^*K*^–values as *G*_*c*_ increases. For the depolarized branch, this shift is much smaller and the green bifurcation line is consequently shorter in the (Δ*N*^*K*^, *V*)–plane.

For low *G*_*c*_, the upper and lower fixed point branch overlap, and the sharp transition from a depolarized state to a polarized state is possible. For higher values there is a gap between the branches and instead of repolarization the system goes into persistent low amplitude oscillations after the upper branch becomes unstable. We have not included the corresponding limit cycles in Fig 6a, however, the time series in Fig 4 shows such oscillations. In Fig 4, the oscillations are unstable, causing the cell to transition to the polarized state. In case of recovery-failure, these small amplitude oscillations persist for the duration of simulations.

Since we understand repolarization as the transition between overlapping fixed point branches, the interesting question is, at what glutamate level this overlap disappears. Above this level, recovery is no longer possible. In Fig 6b, the end of the upper stable fixed point branch and the beginning of the lower branch are shown in the (*G*_*c*_, Δ*N*^*K*^)–plane. As long as the depolarized branch ends after the polarized branch begins, recovery is possible. The critical *G*_*c*_–value is hence at the intersection of the two lines, which occurs near 0.035 mM. If *G*_*c*_ was not a dynamical variable, but a system parameter, this value would be the threshold for recovery failure in SD.

In our simulation of the full system, however, we cannot separate the dynamics of *V* and *G*_*c*_. As *V* decreases, glutamate release slows down and consequently *G*_*c*_ decreases too. On the other hand, an increasing glutamate level depolarizes the neuron, and accordingly the two effects amplify each other. This leads to the glutamate drop from about 0.1 mM to nearly zero at the repolarization point (see Fig 4c). Because of this fast interplay of *V* and *G*_*c*_, the critical value derived in Fig 6b is not an obvious threshold in the full system, but only gives us a rough idea about glutamate levels near the repolarization point.

The fixed point curves in Fig 6a do not approximate the dynamics of the whole system. Moreover, even the fast scale glutamate dynamics of the whole system are only a rough approximation of a real system. Nevertheless, we have learned that critical glutamate levels exist, beyond which the neuron will not repolarize. This observation is based on a model with a parametrical glutamate concentration in the cleft and glutamate coupling through NMDA and AMPA receptors. These parts of our model are based on a more accurate biophysical description than the release and uptake mechanisms of glutamate. Accordingly, we are confident that the effect we have found in Fig 6 is relevant: too much glutamate prevents recovery from SD. In the next section we will provide more insights into this effect.

### Hyperpolarization, recovery, and glutamate

To understand how glutamate interferes with recovery, it is helpful to look at the membrane model, because the first step towards recovery is a change in the membrane state. A given set of ion concentrations determines the reversal potentials *E*_*ion*_ and the pump current *I*_*p*_. These quantities define the membrane model belonging to this ion configuration. We are now interested in the membrane models of the ion configurations around the repolarization point. That means that ion concentrations are now model parameters and we vary them such that we obtain the ion configurations on the depolarized fixed point branch near the repolarization point in Fig 5b. This parameter variation is naturally parametrized by Δ*N*^*K*^.

**Fig 7:**
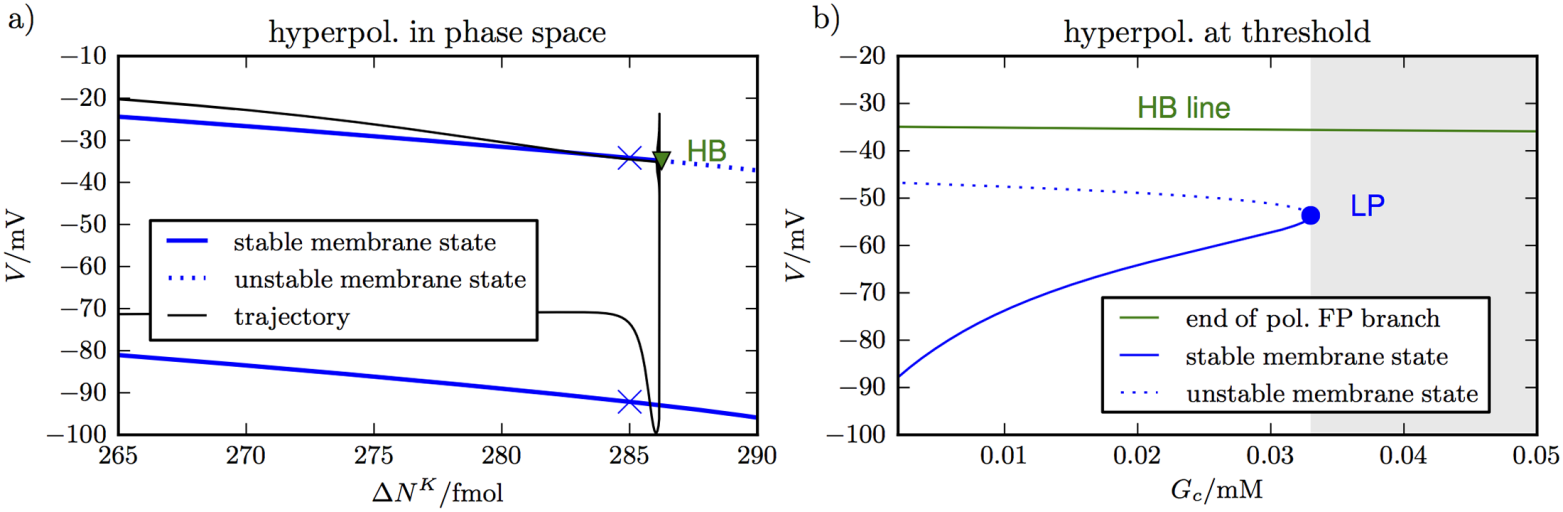
Membrane phase space near repolarization point. Panel **(a)** shows the stable and unstable fixed points of the membrane model as Δ*N*^*K*^ varies for extremely low *G*_*c*_ (set to 0.0001 mM as in Fig 2). There is a stable depolarized and a stable (hyper–)polarized state. At the repolarization point (triangle marker) the depolarized state becomes unstable. The trajectory is guided by the fixed point branches and gets close to the lower branch before ion concentrations adjust and the neuron approaches a level slightly above the lower branch. A de– and a hyperpolarized state close to but before repolarization are marked. The values are given in Table D in S1 Text. Panel **(b)** shows the potentials at the ends of the upper fixed point branches for all *G*_*c*_–values between 0 and 0.05 mM. The other membrane fixed point states, stable hyperpolarization, and an unstable state, are also shown. The stable hyperpolarized state ceases to exist for *G*_*c*_–values higher than 0.32 mM. Beyond this critical value repolarization is no longer possible (shaded region). The critical value is consistent with the value we have derived for the transmembrane model in Fig 6.

The result of this continuation is shown in Fig 7a. There are two fixed points in the membrane model. One is depolarized and coincides with the fixed point of the whole transmembrane model. The other fixed point is in fact hyperpolarized. That is, it is more strongly polarized than *E*_*K*_. At the repolarization point, the depolarized state becomes unstable, while the hyperpolarized state continues to exist. The membrane potential drops very close to this point before ion concentrations and Nernst potentials re–adjust and bring the system to a slightly higher potential.

The existence of a stable hyperpolarized membrane state is what initially drags the membrane potential down and is crucial to the neuron’s recovery. Let us have a closer look at the membrane states and compare the two shortly before the repolarization point. In Fig 7, we have marked the two stable membrane states that exist for Δ*N*^*K*^ = 285 fmol. Some quantities that characterize the membrane state are listed in Table D in S1 Text. The conductances in the depolarized state are dominated by the gated channels which can be seen from $g_{K}\gg g_{K}^{l}$ (and similar for Na^+^). For the hyperpolarized state, the opposite is true and we have $g_{K}\approx g_{K}^{l}$ instead. Accordingly, the hyperpolarized fixed point condition can be approximated as

$$\begin{array}{c} g_{K}^{l}\left(V-E_{K}\right)+g_{Na}^{l}\left(V-E_{Na}\right)+g_{Cl}^{l}\left(V-E_{Cl}\right)=-I_{p}. \end{array}$$ (32)

We can draw the following conclusion from this relation. The pump current *I*_*p*_ is nearly maximal, because we assume a high concentration of extracellular K^+^. Since the leak conductances are rather small, we conclude that the potential difference terms (*V* − *E*_*ion*_) must be sufficiently negative in the polarized fixed point. In particular, the large pump current forces the membrane potential below the K^+^ Nernst potential, which is what we call hyperpolarization. In summary, hyperpolarization is the result of a large pump current and very small conductances. If the leak conductances were not as small, the potential difference terms in Eq (32) would be less negative and depolarization would become weaker. In turn less depolarization will violate the approximation $g_{K}\approx g_{K}^{l}$ and instead *g*_*K*_ would be larger than $g_{K}^{l}$, which leads to even less depolarization. So we have an understanding of repolarization that demonstrates how important it is that the conductances of the neuron collapse strongly enough.

The above consideration only took into account leak conductances and the normal gated ion channels. In addition to that, an elevated glutamate concentration in the cleft implies increased conductances of the NMDA and AMPA receptors as well and the same argument as above holds—the increased conductance implies a less polarized lower fixed point. Again, we increase *G*_*c*_ as a parameter and study its effect. In Fig 7b, we track the hyperpolarized state as we follow the end of the depolarized fixed point branch. For values from 0 mM to 0.0336 mM, there is a stable polarized membrane state when the upper fixed point branch ends. It becomes less polarized with increasing *G*_*c*_ which is consistent with the above reasoning on the importance of collapsing conductances. For very high *G*_*c*_ values, a hyperpolarized membrane state no longer exists and repolarization becomes impossible. The critical value is consistent with the value we derived from the transmembrane model in Fig 6b. In summary, the membrane model teaches us two things. First, a breakdown of neural conductances is needed for the neuron to repolarize sufficiently. Second, over-stimulated synapses can prevent this process and lead to recovery failure.

### Experimental support for model predictions

To confirm model predictions, we recorded SD episodes in layers 2-3 of visual cortex slices from 15-24 days old, male wild type Sprague Dawley rats, both at individual neuron and network levels. To initiate SD, we replaced control ACSF with high KCl (26mM) ACSF (see Methods section). EC and IC electrodes were placed about 500*μ*m apart. In high K^+^ ACSF, SD typically occurred 30-45s after high KCl application. SD in the single cells in control groups started with a rapid depolarization followed by several spikes and a slow return to the resting membrane potential. The majority of cell spiking occurred before maximum depolarization was reached. SD in the EC recording was typically noticeable a few seconds later. SD in single cells typically lasted for 30-180 seconds.

**Fig 8:**
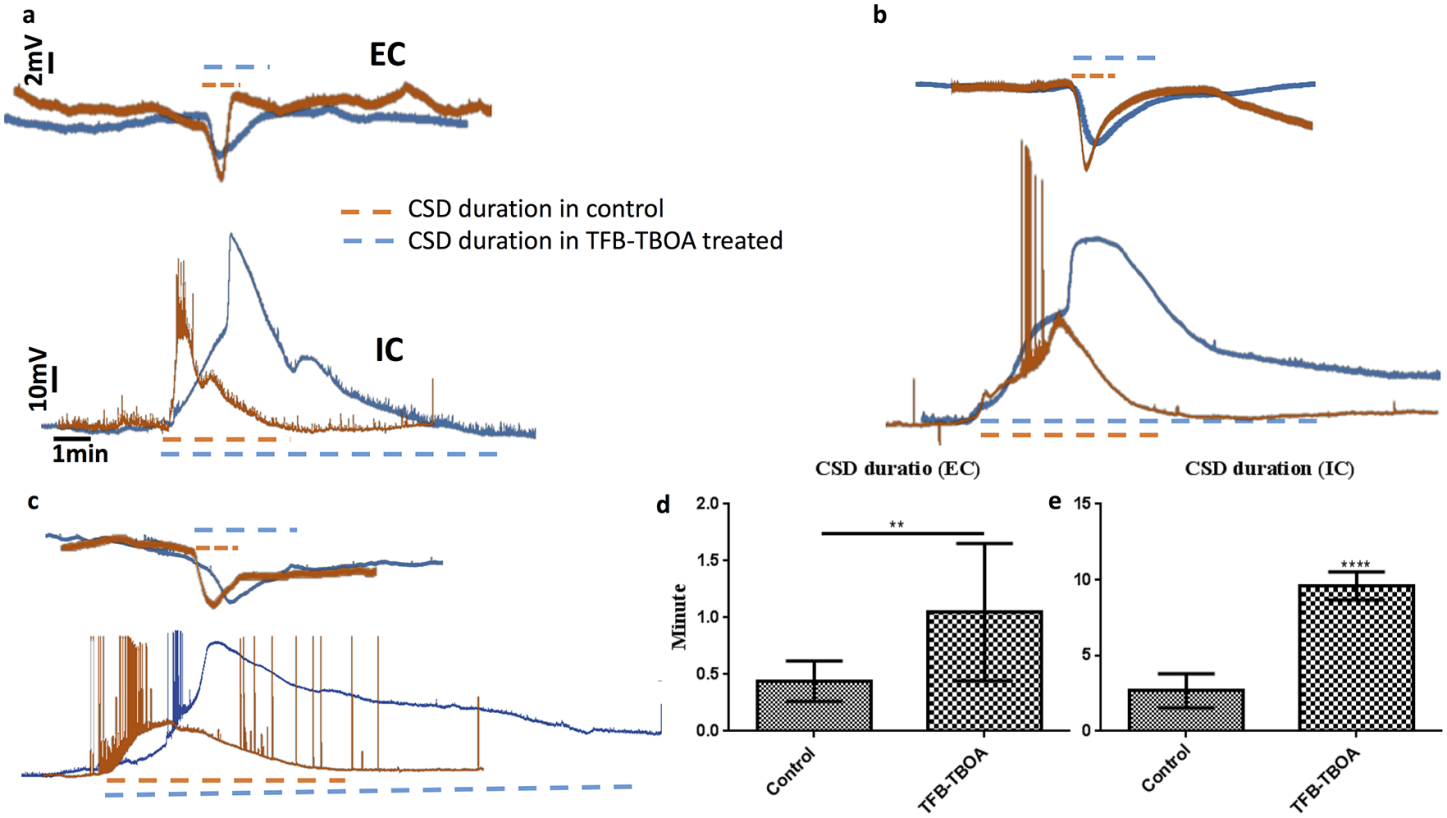
Inhibitor of astrocytic glutamate transporters, TFB-TBOA, significantly increases the duration of SD. Here we show the network and single cell properties of SD in control and TFB-TBOA treated groups. Representative time traces of membrane potential of individual pyramidal neurons (bottom trace) and network level (top trace) in layers 2-3 of visual cortex recorded using whole-cell patch clamp and extracellular recording techniques respectively in control (n = 9) and TFB-TBOA treated groups (n = 9) are shown in panels **(a–c)**. **(d-e)** SD duration defined as the time from the initiation of rapid depolarization in individual neurons and network to the time when the membrane potential repolarizes to its pre-SD value. TFB-TBOA prolonged the duration of SD both at the network level **(d)** (n = 9, p<0.001) and single cell level **(e)** (n = 9, p<0.01).

To evaluate the effect of impaired astrocytic and higher glutamate concentration in the ECS on SD, slices were incubated with 50nM astrocytic glutamate transporter blocker TFB-TBOA for 20 minutes. Fig 8 shows a summary of these experiments. Example traces representing the membrane potential of individual pyramidal cell (bottom traces) and EC recording at the network level (top traces) during SD from control (n = 9) and TFB-TBOA-treated (n = 9) slices are shown in Fig 8a–8c. Unlike control slices where several spikes were observed before the cell entered a depolarization block, TFB-TBOA completely blocked action potentials at the single cell level. The resting membrane potential was not affected by the application of TFB-TBOA.

The mean duration of SD averaged over many events at the network and single cell level is shown in Fig 8d and 8e respectively. SD duration was defined and measured as the time from the initiation of rapid depolarization in individual neurons and network to the time when the membrane potential repolarized to its pre-SD potential value (dashed lines in Fig 8a–8c). In line with model predictions, blocking astrocytic glutamate transporters almost quadruples the duration of SD. It is also worth noticing that single neurons exhibit a stronger depolarization as compared to neurons in control slices. Furthermore, some neurons in control slices exhibit a behavior that is termed as mixed seizure and SD state [94, 44, 43] (see for example lower trace for control cell in Fig 8c), while strong SD was observed in almost all single neurons in the slices treated with TFB-TBOA.

We would like to remark that, although not exactly the same, the average SD duration given by the model is comparable to experimentally observed values. A 100% glutamate uptake in the model gives over a minute long SD as compared to ∼ 2.5 minutes long SD in control slices. This difference could be due to the higher K^+^ in the perfusion solution used in the experiment as compared to the model. It could also be due to our overestimation of the glutamate uptake (or underestimation of glutamate release) as lower uptake leads to longer SDs. Application of TFB-TBOA increases the duration of SD to almost 10 minutes. Decreasing the glutamate uptake by transporters to about 17.5% of the control value in the model leads to a comparable (about 4 times) increase in the duration of SD as compared to control simulation. Reducing the uptake to 16% of the control value will lead to SD duration comparable to the observed values. We would like to point out that TFB-TBOA targets only astrocytic glutamate transporters, while the reduction in glutamate uptake in the model applies to both neuron and astrocyte. So, the application of TFB-TBOA does not necessarily mean the complete inhibition of glutamate uptake. Given that glia cell has about eight times more binding sites for glutamate and has about half the surface area available for uptake (see Diffusion and Glutamate Uptake section) as compared to neuron, there would still be roughly one-fourth glutamate transporters intact (ignoring other complications due to morphology, ion concentration dynamics etc.) even in the presence of TFB-TBOA.

We also observed time-dependent changes in the neuronal properties due to TFB-TBOA. Action potentials were evoked by current pulses and studied under the current-clamp conditions (n = 9). TFB-TBOA (50nM) increased the cell membrane resistance from 150MΩ to 260MΩ (p<0.05) after 10 minutes. Current pulses evoked fewer APs (7 versus 11 on average) in slices pretreated with 50nM TFB-TBOA (p<0.05) (0-100pA current injection). After 20min treatment with 50nM TFB-TBOA, the AP threshold increased from -45mV to -32mV (p<0.05), while the amplitude of AP decreased from 80mV to 30mV (p<0.001). AP amplitude was measured as the voltage difference between the threshold and the peak value of the membrane potential during the AP. We believe that these changes in the neuronal properties could be due to the reduction in Na^+^ influx due to TFB-TBOA. In line with this argument, Bozzo et al. [95] demonstrated the inhibitory effects of TFB-TBOA on astrocytic Na^+^ responses to glutamate. They also claimed that TFB-TBOA has no effect on the membrane properties of cultured cortical neurons recorded in the whole-cell patch clam. Recently, Hosseini-Zare et al [90] on the other hand, claimed that fast voltage-gated Na^+^ currents are reduced by TFB-TBOA. Whether the reduction in Na^+^ currents is caused by a direct interaction of TFB-TBOA with fast voltage-gated Na^+^ channels, through inhibition of Na^+^ cotransport (notice that three Na^+^ are cotransported with one glutamate molecule), or through some other mechanism is not entirely clear. While our model includes the inhibition of Na^+^ cotransport through glutamate transporters, incorporating the effect of TFB-TBOA on fast voltage-gated Na^+^ currents, if proven unequivocally, in the model is beyond the scope of this study and will be investigated in the future.

## Discussion

Significant experimental and clinical data suggest that SD is involved in numerous brain pathologies including migraine, stroke, subarachnoid hemorrhage, and traumatic brain injury [13, 12, 3, 4]. The initiation, propagation, sustainment, and termination of SD involve immense changes in many molecular and cellular pathways shaping the interplay between neurons, extracellular space, glial cells, and vasculature. What complicates things further is that most of these modifications are dependent on each other, and some may have biphasic role [96]. For example, over-activation of NMDA receptors in the early phase of stroke is detrimental, but in delayed phase, they might mediate neuroprotection through neuroplasticity [97]. That is probably why all trials testing NMDA antagonists for stroke treatment have failed [96, 98]. In clinical trials, SD episodes were discontinued in two patients treated with ketamine on one hand [29], while on the other hand, a cluster of SD occurred in another patient despite the presence of ketamine [99]. Thus a complete understanding of SD and finding clinically useful therapeutic interventions for the related pathologies hinge on elucidating this wide array of changes. However, the current experimental and clinical tools are too limited to simultaneous investigate all these changes, which necessitates physiologically relevant detailed computational models.

In this paper, we developed a comprehensive model that incorporates many key elements involved in the dynamics of SD including: neuronal membrane potential dynamics, ion concentration dynamics in neuron, extracellular space, and glial cell, ion exchange with vasculature, swelling of neuron and glia, and detailed formalism of glutamate release and uptake processes. Although, we explore the effect of glutamate uptake and extracellular levels on the initiation, sustainment, and termination of SD, our model allows us to investigate the role of all these factors in the dynamics of SD simultaneously or one by one.

Our results show that glutamate signaling plays a key role in the dynamics of SD since impaired glutamate uptake prolongs the duration of SD and leads to significant neuronal and glial swelling. Reducing glutamate uptake by transporters below 16% of the control value leads to the failure of cell’s recovery from SD. We verified this prediction experimentally by showing that SD in layers 2-3 of visual cortex from 15-24 days old rats are significantly prolonged by inhibiting glial glutamate uptake using TFB-TBOA. Our computational results are also consistent with a recent study, which showed that 0.5 and 1 mM TBOA prolonged SD by 148% and 426% respectively [28]. A respective increase of 167% and 374% in glutamate concentration was observed in the same experiments. Our result is also in line with conclusions from *in vivo* and *in vitro* studies in Ref. [80], where the elimination of glial glutamate transporters were shown to lead to tonic increase in extracellular glutamate, resulting in widespread swelling and neuronal degeneration. Furthermore, increasing the expression of glial glutamate transporter EAAT2 through application of *β*-lactam antibodies significantly reduced extracellular glutamate in animal studies and protected against ischemic injury and neurodegeneration [33, 32].

We would like to remark that the pattern of neuronal and glial swelling, and the dynamics of various ion concentrations observed in our model explains several experimental observations. We skip such details here and refer the interested reader to our recent work for a detailed discussion about these observations and the role of swelling in ischemic injury [56, 53]. Furthermore, the dysfunction of glial glutamate transporters is also implicated in other acute and chronic neurological disorders including amyotrophic lateral sclerosis [100], brain tumors [101], epilepsy [102, 103], Alzheimer’s disease [104], and motor discoordination [105]. Our approach can be adopted to quantify the role of different pathways involved in glutamate dynamics in these conditions.

As mentioned above, our model skips several factors that could be key for glutamate homeostasis. For example, glutamate flux through astrocytic glutamate transporters reverses direction in the presence of high EC K^+^ or high IC glutamate concentration [106, 107]. Thus, during SD where K^+^ and glutamate are both high, reversal of glutamate transport in astrocytes could become an additional source of EC glutamate build-up. The blockade of GLT transporters during SD in our experiments may have prevented glutamate transport reversal and caused glutamate to be trapped inside the glia, decreasing EC glutamate levels. Under different experimental conditions, however, it was observed that a different glutamate transporter blocker (D,L-threo-beta-hydroxyaspartate (THA)) increased the amount of depolarization and duration of SD in the presence of high K^+^ [108]. The changes in the single cell properties observed in our experiments are also ignored in our model. Furthermore, our study is concerned mainly with the behavior of a single neuron during SD. Investigating the effect of changes in glutamate homeostasis on the spatial spread of SD will require a network model. Incorporating these key factors in the model is beyond the scope of the current manuscript and is the subject of our future studies.

We would also like to point out that there are different types of SDs with different features and probably different mechanisms for induction and propagation [109, 3]. Our study focuses on SD caused by K^+^ perfusion and OGD. SD due to OGD is particularly relevant for stroke. Whether glutamate is necessary for propagation of all kinds of SDs is still debated. Interestingly, very little glutamate diffuses out of the cleft when uptake is not impaired in our model, consistent with a glutamate-independent propagation of SD in our modeling conditions.

To conclude, by combining an established semi–phenomenological neuron–glia description and first physical principles in a consistent way, we have developed a physiologically relevant, comprehensive model that incorporates many key components involved in the dynamics of SD. This new mathematical framework describes many aspects of neuronal membrane, ion concentration dynamics, cell swelling, and glutamate dynamics during SD accurately and provides deep insights into the mechanisms through which glutamate interferes with neuronal recovery from SD. We present strong experimental evidence in support of our study, and emphasize that most of our explanations come from general physical principles and biophysical reasoning. The theory is general and the components included are key to both normal and pathological brain function. Accordingly, we claim that our approach can be used as a future guide to investigate the role of ion concentrations, ion exchange with glia and blood vessels, cell swelling, and glutamate dynamics in other brain pathologies and normal brain function. For example, to investigate glutamate homeostasis in regular neuronal firing, one would assume that only a few synapses are involved in the release of glutamate unlike SD where we assume all 10,000 synapses releasing glutamate. Similarly, to investigate these variables in seizures induced by high *K*^+^, higher *K*^+^ concentration in the bath, *K*_*bath*_ (typically 8mM) should be used. Furthermore, the additional currents involved in the specific neuronal behavior in question should be included in the membrane potential ion concentrations dynamics.

## Supporting information

S1 Text Equations for neuronal membrane potential and ion dynamics, modifications to these equations due to the incorporation of glutamate homeostasis, and the morphology considered in the model. (PDF)

S2 Text The AUTO code reproducing the key results in this paper. (ZIP)
