# Supplementary material for: The role of glutamate in neuronal ion homeostasis: A case study of spreading depolarization
Source: PLoS Comput Biol. 2017 Oct 12;13(10):e1005804. doi: 10.1371/journal.pcbi.1005804 (PMC5655358; doi:10.1371/journal.pcbi.1005804)
Supplement: S1 Text — (PDF) [file pcbi.1005804.s001.pdf]

S1 Text: The Role of Glutamate in Neuronal Ion Homeostasis: A Case Study of Spreading Depolarization

Niklas Hübel [1], Mahshid S Hosseini-Zare[2], Jokūbas Žiburkus [2], Ghanim Ullah [1,*]
[1]Department of Physics, University of South Florida,
Tampa, FL 33620, USA,
[2]Department of Biology and Biochemistry, University of Houston,
Houston, TX 77204, USA

Standard Model for Neural Ion Dynamics

Rate equations for the membrane potential of the neuron, gating variables for K^+ and Na^+ channels, ion concentrations inside the neuron, glia, and ECS, and volumes of the neuron, glia cell, and ECS are based on our previous work [1, 2, 3, 4, 5, 6, 7, 8, 9, 10, 11, 12]. Here we describe these equations together with the modifications due to the inclusion of glutamate dynamics, and the morphology used in this model.

Membrane Model

We refer to the rate equations for the membrane potential and the gating dynamics as the membrane model. It is a variant of the classical Hodgkin–Huxley (HH) description of the neural membrane [13, 14]. The membrane has a capacitance C_m and carries a potential V, which follows a Kirchhoff law and is governed by the K^+, Na^+, and Cl^- ion currents I_K, I_{Na}, and I_{Cl}, respectively. In the rate equations below a pump current I_p is also included. It has an important role in the ion dynamics (see below).

There are leak ion channels and voltage–gated channels that depend on the gating variables n (K^+ activation), m (Na^+ inactivation), and h (Na^+ activation). The gating dynamics are given by the HH exponential functions α_x and β_x (for $x \in \{n, m, h\}$). The full membrane

model reads

$$\frac{\mathrm{d}V}{\mathrm{d}t} = -\frac{1}{C_m}(I_{Na} + I_K + I_{Cl} + I_p) \ , \tag{1S}$$

$$\frac{\mathrm{d}n}{\mathrm{d}t} = \phi\Big(\alpha_n(1-n) - \beta_n n\Big) \ , \tag{2S}$$

$$\frac{\mathrm{d}h}{\mathrm{d}t} = \phi\Big(\alpha_h(1-h) - \beta_h h\Big) \ , \tag{3S}$$

with the conventional timescale parameter ϕ, and an adiabatic approximation for the extremely fast m–gate:

$$m = m_\infty = \frac{\alpha_m}{\alpha_m + \beta_m} \tag{4S}$$

The voltage–dependent exponential functions are

$$\alpha_n = \frac{0.01(V+34)}{1-\exp(-(V+34)/10)} \ , \tag{5S}$$

$$\beta_n = 0.125\exp(-(V+44)/80) \ , \tag{6S}$$

$$\alpha_m = \frac{0.1(V+30)}{1-\exp(-(V+30)/10)} \ , \tag{7S}$$

$$\beta_m = 4\exp(-(V+55)/18) \ , \tag{8S}$$

$$\alpha_h = 0.07\exp(-(V+44)/20) \ , \tag{9S}$$

$$\beta_h = \frac{1}{1+\exp(-(V+14)/10)} \ . \tag{10S}$$

These equations were originally adopted from [14] and have been used extensively by us and other groups. Nevertheless, we have tested in the past that states like seizure, SD, and anoxic depolarization are robust in the face of different types of formalisms for ion channels including the Goldman-Hodgkin-Katz formalism [9].

The currents I_{ion} (for $ion \in \{K, Na, Cl\}$) are all of the form

$$I_{ion} = g_{ion}(V - E_{ion}) \ . \tag{11S}$$

with conductances g_{ion} that are a combination of leak and gated parts. Leak conductances and maximal conductances of gated channels are denoted by a superscript l and g, respec-

tively:

$$g_K = g_K^l + g_K^g n^4 \ , \tag{12S}$$

$$g_{Na} = g_{Na}^l + g_{Na}^g m^3 h \ , \tag{13S}$$

$$g_{Cl} = g_{Cl}^l \ . \tag{14S}$$

The Nernst potentials E_{ion} are given by the ion concentrations $ion_{i/e}$ in the intra–/extracellular space (ICS and ECS), and the ion valence z_{ion}:

$$E_{ion} = \frac{26.64}{z_{ion}} \ln(ion_e/ion_i) \tag{15S}$$

The coefficient 26.64 mV can be derived from the ideal gas constant, the absolute temperature, and Faraday's constant. In addition to these currents we will also include Na^+ and K^+ currents through AMPA and NMDA receptor gates. Also glutamate uptake by the neuron comes with ion cotransport which will be included as well (see below). The conductances and other parameters defining the membrane model are given in Table A.

We would like to remark that the Hodgkin-Huxley formalism uses linear relationships for current and voltage (Ohm's law). Nevertheless, it is well known that the actual current flowing through ion-selective permeability channels in the neuronal membrane is nonlinear, rectifying [15], and can be more accurately accounted for using the Goldman-Hodgkin-Katz (GHK) equations [16]. However, we have previously shown that the two formalisms result in qualitatively same dynamics under extreme conditions such as seizures and SD [9].

Ion Dynamics, Ion Regulation, and Osmosis

The above introduced currents induce ion fluxes. The changes in the intracellular ion amounts N_i^{ion} due to these currents follow from converting currents to fluxes by a factor

$$\gamma = \frac{A_m}{F} \tag{16S}$$

that depends on the membrane surface area A_m and Faraday's constant F:

$$\frac{\mathrm{d}N_i^{Na}}{\mathrm{d}t} = -\gamma(I_{Na} + 3I_p) \ , \tag{17S}$$

$$\frac{\mathrm{d}N_i^{K}}{\mathrm{d}t} = -\gamma(I_{K} - 2I_p) \ , \tag{18S}$$

$$\frac{\mathrm{d}N_i^{Cl}}{\mathrm{d}t} = \gamma I_{Cl} \ . \tag{19S}$$

The pump terms I_p account for exchange of intracellular Na^+ for extracellular K^+ at a 3/2 ratio. Pumping gets stronger when extracellular K^+ (K_e) or intracellular Na^+ (Na_i) concentrations increase and has a maximal turnover rate ρ:

$$I_p = \rho\left(1 + \exp\left(\frac{15 - Na_i}{3}\right)\right)^{-1}\left(1 + \exp\left(5.5 - K_e\right)\right)^{-1} \tag{20S}$$

Such a straightforward extension of the membrane model to include ion dynamics is found in many computational studies on SD, stroke and seizure–like brain dynamics [17, 1, 2, 3, 4, 6, 9, 18, 11, 19, 20]

Electroneutrality is important symmetry and is conserved in all compartments. Similarly, the model also ensures mass conservation in the system. Electroneutrality of the intracellular space implies that the intracellular charge concentration

$$Q_i := Na_i + K_i - Cl_i = Na_i^0 + K_i^0 - Cl_i^0 \tag{21S}$$

is constant. Where Na_i, K_i, and Cl_i are intracellular concentrations of Na^+, K^+, and Cl^+ respectively. Accordingly Na_i, K_i and Cl_i are not independent, and one of the rate Eqs. (17S)–(19S) can be replaced by simply solving Eq. (21S) for that variable. Note that initial physiological resting conditions are denoted by a superscript 0.

Also mass conservation holds and ion concentrations in the ECS follow from values in the ICS by solving the following constraint equations:

$$Na_i\omega_i + \mathrm{Na}_e\omega_e = Na_i^0\omega_i + \mathrm{Na}_e^0\omega_e \ , \tag{22S}$$

$$K_i\omega_i + \mathrm{K}_e\omega_e = K_i^0\omega_i + \mathrm{K}_e^0\omega_e + \Delta\mathrm{N}^K \ , \tag{23S}$$

$$Cl_i\omega_i + \mathrm{Na}_e\omega_e = Cl_i^0\omega_i + \mathrm{Cl}_e^0\omega_e \ . \tag{24S}$$

The extracellular ion amounts follow from mass conservation. Thus we have

$$N_e^{Na,0} + N_i^{Na,0} = N_e^{Na} + N_i^{Na} - \Delta N_{glia}^{Na} \ , \tag{25S}$$

$$N_e^{K,0} + N_i^{K,0} = N_e^{K} + N_i^{K} - \Delta N_{glia}^{K} - \Delta N_{bath}^{K} \ , \tag{26S}$$

$$N_e^{Cl,0} + N_i^{Cl,0} = N_e^{Cl} + N_i^{Cl} - \Delta N_{glia}^{Cl} \ . \tag{27S}$$

The difference terms ΔN_{glia}^{ion} and ΔN_{bath}^{K} represent ion exchange with glia cells and an external K^+ bath, respectively. The formulation in terms of these differences has been first proposed in Ref. [2], where it was also pointed out that particle exchange with external reservoirs is much slower than transmembrane ion fluxes (see below).

Diffusive coupling to the bath is driven by the concentration difference between the ECS and the bath:

$$\frac{\mathrm{d}\Delta N_{bath}^{K}}{\mathrm{d}t} = \lambda(K_e - K_{bath}) \tag{28S}$$

This coupling scheme is defined by the bath coupling strength λ and the K^+ concentration in the bath K_{bath}. We are going to model an experimental procedure where brain slices are perfused with a high K^+ solution to initiate SD. In the model this can be done by setting K_{bath} to a higher value.

Glial K^+ buffering is modelled by assuming a K_e–dependent rate of K^+ uptake

$$\lambda^{up} = \lambda_1 \left(1.0 + \exp\left(\frac{5.5 - K_e}{2.5} \right) \right)^{-1} \tag{29S}$$

and a constant small re–release rate λ^{rel}:

$$\frac{\mathrm{d}\Delta N_{glia}^{K}}{\mathrm{d}t} = -\lambda^{rel} + \lambda^{up} \, \frac{\Delta N_{glia}^{K,max} - \Delta N_{glia}^{K}}{\Delta N_{glia}^{K,max}} \tag{30S}$$

Note that we assume a limited uptake capacity $\Delta N_{glia}^{K,max}$. Glia swelling will be important and it is hence necessary to include all glial ion fluxes (see below). The fluxes of Na^+ and Cl^- can be approximated by

$$\Delta N_{glia}^{Na} = -0.2 \Delta N_{glia}^{K} \ , \tag{31S}$$

$$\Delta N_{glia}^{Cl} = 0.8 \Delta N_{glia}^{K} \ . \tag{32S}$$

This approximation is motivated by experimental data on glial ion channels and it guarantees electroneutrality [4]. Both bath coupling and the glial buffering model have been used in previous studies [17, 4]. The relationship between ΔN_{glia}^{Na} and ΔN_{glia}^{K} was derived and justified previously [4].

During SD neural and glial swelling reduce the ECS to about 25% of its normal value. This affects all extracellular particle concentrations dramatically. Swelling is driven by osmosis. Ion fluxes create osmotic gradients and cellular volumes adjust to re–establish osmotic equilibrium between ICS, ECS, and glia. Let us denote the respective volumes by $\omega_{i/e/g}$ and the total amounts of matter by $N_{i/e/g}$, for example

$$N_i = N_i^{Na} + N_i^{K} + N_i^{Cl} + N_i^{A} + N_i^{X} \ . \tag{33S}$$

This expression contains additional types of particles, namely impermeant anions N_i^A and impermeant neutral matter N_i^X. They ensure initial osmotic equilibrium. The equilibrium is defined by the condition

$$\frac{N_i}{\omega_i} = \frac{N_e}{\omega_e} = \frac{N_g}{\omega_g} = \frac{N_{tot}}{\omega_{tot}} \quad \Rightarrow \quad \omega_{i/e/g} = N_{i/e/g}\frac{\omega_{tot}}{N_{tot}} \ , \tag{34S}$$

where $N_{tot} = N_i + N_e + N_g$ and $\omega_{tot} = \omega_i + \omega_e + \omega_g$. This relation shows that the volume of a compartment grows and shrinks with the amount of particles in it, which is why events with extreme ion fluxes sometimes can lead to strong cellular swelling. Strictly speaking the above expression for $\omega_{i/e/g}$ only gives the equilibrium volumes, which can differ from the actual volumes, because it takes time for the cells to swell and recover from swelling. However, it can be shown that for neurons and glia cells volume changes happen nearly instantaneously [4] and Eq. (34S) is satisfied at all times. We remark that the glial ion content is not modeled explicitly, but instead we assume an initial content N_g^0 such that the glia cell is in balance with the ECS, i.e., $N_g^0/\omega_g^0 = N_e^0/\omega_e^0$. The content at later times is then

$$N_g = N_g^0 + \Delta N_{glia}^{Na} + \Delta N_{glia}^{K} + \Delta N_{glia}^{Cl} \ . \tag{35S}$$

Volume changes are sometimes included in SD models. The volume model we chose is not the commonly used one, but it seems physically more consistent than the more phenomenological descriptions found in Refs. [21, 20, 6, 9] (for details see Ref. [4]).

Modifications to the Membrane Potential and Ion Dynamics Due to Glutamate Dynamics

Please read "Glutamate–Related Processes" section in the main text first for the following equations to make sense. Uptake of glutamate goes along with ion cotransport [22]. For the neuron, one molecule of glutamate is accompanied by three Na^+ and one Cl^-, while it releases one K^+. These contributions can be converted to the cotransport currents

$$I_{Na}^{co} = \frac{3}{\gamma}(v_{c\to n} + v_{e\to n}) \ , \tag{36S}$$

$$I_{Cl}^{co} = \frac{1}{\gamma}(v_{c\to n} + v_{e\to n}) \ , \tag{37S}$$

$$I_{K}^{co} = \frac{-1}{\gamma}(v_{c\to n} + v_{e\to n}) \ . \tag{38S}$$

These and the AMPA and NMDA currents must be added to the rate equation for the membrane potential and those for the ion changes, i.e. we need to replace

$$I_{Na} \longrightarrow I_{Na} + I_{Na}^{AMPA} + I_{Na}^{NMDA} + I_{Na}^{co} \tag{39S}$$

$$I_{K} \longrightarrow I_{K} + I_{K}^{AMPA} + I_{K}^{NMDA} + I_{K}^{co} \tag{40S}$$

$$I_{Cl} \longrightarrow I_{Cl} + I_{Cl}^{co} \tag{41S}$$

in Eqs. (1S) and (17S–19S).

Morphology

Most single–compartment models for neural ion dynamics assume a nearly spherical shape which resembles a soma rather than a whole neuron. However, glutamate dynamics mostly involves processes at the dendritic terminals, so this assumption is not appropriate here. For glutamate dynamics, it is relevant that how many synapses are there, how large the ECS is and how much membrane surface area is available to take up glutamate from the ECS back into the cells.

Table B compares values for a soma and a whole neuron including the dendrites. We use the values of the latter. Moreover we assume glia cells of equal size and an extracellular volume that corresponds to about 15% of the whole tissue [17, 23, 24]. This is achieved by assuming volume ratios 3: 1: 3 between ICS, ECS, and glia, respectively. Note that the values given in Table B are initial resting values and will change during SD.

References

1. Hübel N, Schöll E, Dahlem MA (2014) Bistable dynamics underlying excitability of ion homeostasis in neuron models. PLoS Comp Biol 10: e1003551.

2. Hübel N, Dahlem MA (2014) Dynamics from seconds to hours in Hodgkin–Huxley model with time–dependent ion concentrations and buffer reservoirs. PLoS Comp Biol 10: e1003941.

3. Hübel N, Andrew DR, Ullah G (2016) Large extracellular space leads to neuronal susceptibility to ischemic injury in Na^+/K^+ pumps-dependent manner. J Comput Neurosci 40: 177–192.

4. Hübel N, Ullah G (2016) Anions govern cell volume: A case study of relative astrocytic and neuronal swelling in spreading depolarization. PLoS ONE 11: e0147060.

5. Ullah G, Cressman Jr JR, Barreto E, Schiff SJ (2009) The influence of sodium and potassium dynamics on excitability, seizures, and the stability of persistent states: Ii. network and glial dynamics. J Comput Neurosci 26: 171–183.

6. Ullah G, Wei Y, Dahlem MA, Wechselberger M, Schiff SJ (2015) The role of cell volume in the dynamics of seizure, spreading depression, and anoxic depolarization. PLoS Comp Biol 11: e1004414.

7. Ullah G, Schiff SJ (2010) Assimilating seizure dynamics. PLoS Comput Biol 6: e1000776.

8. Ullah G, Schiff SJ (2009) Tracking and control of neuronal Hodgkin-Huxley dynamics. Phys Rev E 79: 040901.

9. Wei Y, Ullah G, Schiff SJ (2014) Unification of neuronal spikes, seizures, and spreading depression. J Neurosci 34: 11733–11743.

10. Wei Y, Ullah G, Ingram J, Schiff SJ (2014) Oxygen and seizure dynamics: II. Computational modeling. J Neurophysiol 112: 213–223.

11. Cressman Jr JR, Ullah G, Ziburkus J, Schiff SJ, Barreto E (2009) The influence of sodium and potassium dynamics on excitability, seizures, and the stability of persistent states: I. single neuron dynamics. J Comput Neurosci 26: 159–170.

12. Cressman Jr JR, Ullah G, Ziburkus J, Schiff SJ, Barreto E (2011) Erratum to: The influence of sodium and potassium dynamics on excitability, seizures, and the stability of persistent states: I. single neuron dynamics. J Comput Neurosci 30: 781.

13. Hodgkin AL, Huxley AF (1952) A quantitative description of membrane current and its application to conduction and excitation in nerve. J Physiol 117: 500–544.

14. Gutkin BS, Laing CR, Colby CL, Chow CC, Ermentrout GB (2001) Turning on and off with excitation: The role of spike–timing asynchrony and synchrony in sustained neural activity. J Comp Neurosci 11: 121–134.

15. Hille B, et al. (2001) Ion channels of excitable membranes, volume 507. Sinauer Sunderland, MA.

16. Hodgkin AL, Katz B (1949) The effect of sodium ions on the electrical activity of the giant axon of the squid. J Physiol 108: 37-77.

17. Kager H, Wadman WJ, Somjen GG (2000) Simulated seizures and spreading depression in a neuron model incorporating interstitial space and ion concentrations. J Neurophysiol 84: 495–512.

18. Barreto E, Cressman JR (2010) Ion concentration dynamics as a mechanism for neural bursting. J Biol Phys 37: 361–373.

19. Zandt BJ, ten Haken B, van Dijk JG, van Putten MJ (2011) Neural dynamics during anoxia and the "wave of death". PLoS ONE 6: e22127.

20. Yao C, Zhan M (2010) Signal transmission by vibrational resonance in one-way coupled bistable systems. Phys Rev E 81: 061129.

21. Kager H, Wadman WJ, Somjen GG (2002) Conditions for the triggering of spreading depression studied with computer simulations. J Neurophysiol 88: 2700.

22. Benarroch EE (2010) Glutamate transporters—diversity, function, and involvement in neurologic disease. Neurology 74: 259–64.

23. Azevedo FA, Carvalho LR, Grinberg LT, Farfel JM, Ferretti RE, et al. (2009) Equal numbers of neuronal and nonneuronal cells make the human brain an isometrically scaled–up primate brain. J Comp Neurol 513: 532–541.

24. Ascoli GA, Donohue DE, Halavi M (2007) Neuromorpho.org: a central resource for neuronal morphologies. J Neurosci 27: 9247–9251.

Table A: Parameters for membrane and ion dynamics, and initial concentrations.

Name	Value & unit	Description
C_m	1 μF/cm^2	membrane capacitance
ϕ	3/msec	gating time scale parameter
g^l_{Na}	0.0135 mS/cm^2	Na^+ leak cond.
g^g_{Na}	100 mS/cm^2	max. gated Na^+ cond.
g^l_K	0.05 mS/cm^2	K^+ leak cond.
g^g_K	40 mS/cm^2	max. gated K^+ cond.
g^l_{Cl}	0.05 mS/cm^2	Cl^- leak cond.
F	96,485 C/mol	Faraday's constant
ρ	6.46 μA/cm^2	max. pump current
λ	1e–4 /msec	bath coupling strength
K_{bath}	4 mM	K^+ con. in bath
λ_1	1.44e–2 mM/msec	glial K^+ uptake parameter
λ^{rel}	5.1e–3 mM/msec	glial K^+ release rate
$\Delta N^{K,max}_{glia}$	350 fmol	K^+ uptake capacity of glia cell
Na^0_i	15 mM	init. conc. of Na^+ in ICS
Na^0_e	144 mM	init. conc. of Na^+ in ECS
K^0_i	140 mM	init. conc. of K^+ in ICS
K^0_e	4 mM	init. conc. of K^+ in ECS
Cl^0_i	9 mM	init. conc. of Cl^- in ICS
Cl^0_e	130 mM	init. conc. of Cl^- in ECS
A_i	146 mM	init. conc. of imperm. anions in ICS
A_e	18 mM	init. conc. of imperm. anions in ECS
X_i	5 mM	init. conc. of imperm. neutral matter in ICS
X_e	19 mM	init. conc. of imperm. neutral matter in ECS
M_g	315 mM	init. conc. of all matter in glial compartment

Table B: Morphological parameters.

Name	Value & unit	Description
ω_i	$\sim 2{,}000\ \mu m^3$	soma volume
$A_m^{(n)}$	$\sim 900\ \mu m^2$	soma membrane surface area
ω_i	$7{,}500\ \mu m^3$	volume of whole neuron
ω_g	$7{,}500\ \mu m^3$	glia volume (equal size as neuron)
ω_e	$2{,}500\ \mu m^3$	ECS volume ($\sim$ 15% of whole tissue)
$A_m^{(n)}$	$18{,}000\ \mu m^2$	neural membrane surface area
$A_m^{(g)}$	$18{,}000\ \mu m^2$	glial membrane surface area

Table C: Parameters for glutamate–related processes.

Name	Value & unit	Description
V_{cr}	-50 mV	critical potential parameter
V_{hi}	50 mV	high potential parameter
R_{max}	1.50e–5 fmol/msec	maximal release rate
N_{syn}	5000	number of synapses
N^G_{max}	10 fmol	glutamate available for signaling
α_{NMDA}	0.072 /(mM $\times$ msec)	receptor gating constant
β_{NMDA}	0.0066 /msec	receptor gating constant
α_{AMPA}	1.1 /(mM $\times$ msec)	receptor gating constant
β_{AMPA}	0.19 /msec	receptor gating constant
$\overline{g}_{NMDA}$	0.139 mS/cm^2	max. cond. of NMDA rcpt. channel
$\overline{g}_{AMPA}$	0.486 mS/cm^2	max. cond. of AMPA rcpt. channel
[Mg^{2+}]	1.2 mM	external [Mg^{2+}] concentration
r	100 nm	cleft radius
h	20 nm	cleft height
ω_c	1.26e–3 μm^3	cleft volume
ω_{en}	3.77e–3 μm^3	volume within envelope
A_σ	6.3e–3 μm^2	flux cross section area
D_G	0.3 μm^2/msec	glutamate diffusion coefficient
Δx	20 μm	distance from cleft to stationary extrac. glutamate conc.
$v^{max}_{c\to n}$	0.03 mM/msec	maximal glutamate uptake rate (cleft to neuron)
k_m	0.03 mM	equilibration constant for glutamate uptake
k_{rec}	0.001 fmol/msec	glutamate recycling rate

Table D: Polarized vs depolarized membrane state.

Name	Value & unit	Description
E_K	-40.5 mV	K^+ Nernst potential
E_{Na}	-22.1 mV	Na^+ Nernst potential
E_{Cl}	-34.2 mV	Cl^- Nernst potential
I_p	6.46 μA/cm^2	pump current
V	-34.2 mV	depolarized potential
g_K	2.045 mS/cm^2	K^+ cond. in depolarized state
g_{Na}	1.603 mS/cm^2	Na^+ cond. in depolarized state
V	-92.1 mV	hyperpolarized potential
g_K	0.050 mS/cm^2	K^+ cond. in hyperpolarized state
g_{Na}	0.0135 mS/cm^2	Na^+ cond. in hyperpolarized state
